# CD4^+^ T cell–innate immune crosstalk is critical during *Staphylococcus aureus* craniotomy infection

**DOI:** 10.1172/jci.insight.183327

**Published:** 2025-02-24

**Authors:** Gunjan Kak, Zachary Van Roy, Rachel W. Fallet, Lee E. Korshoj, Tammy Kielian

**Affiliations:** Department of Pathology, Microbiology, and Immunology, University of Nebraska Medical Center, Omaha, Nebraska, USA.

**Keywords:** Immunology, Infectious disease, Bacterial infections, Innate immunity, T cells

## Abstract

Access to the brain for treating neurological sequalae requires a craniotomy, which can be complicated by infection. *Staphylococcus aureus* accounts for half of craniotomy infections, increasing morbidity in a medically fragile patient population. T cells preferentially traffic to the brain during craniotomy infection; however, their functional importance is unknown. Using a mouse model of *S*. *aureus* craniotomy infection, CD4^+^ T cells were critical for bacterial containment, as treatment of WT animals with anti-CD4 exacerbated infection that was similar to phenotypes in *Rag1*^–/–^ mice. Single-cell RNA-Seq (scRNA-Seq) revealed transcriptional heterogeneity in brain CD3^+^ infiltrates, with CD4^+^ cells most prominent that displayed Th1- and Th17-like characteristics, and adoptive transfer of either subset in *Rag1*^–/–^ animals during early infection prevented *S*. *aureus* outgrowth. scRNA-Seq identified a robust IFN signature in several innate immune clusters, and examination of cell-to-cell interactions revealed extensive T cell crosstalk with monocytes/macrophages that was also observed in human craniotomy infection. A cooperative role for Th1 and Th17 responses was demonstrated by treatment of *Ifng*^–/–^ mice with IL-17A neutralizing antibody that recapitulated phenotypes in *Rag1*^–/–^ animals. Collectively, these findings implicate Th1- and Th17-mediated proinflammatory responses in shaping the innate immune landscape for *S*. *aureus* containment during craniotomy infection.

## Abstract 2

Immune cell crosstalk during craniotomy infection is shaped by T lymphocytes that is important for restricting bacterial growth.

## Graphical abstract

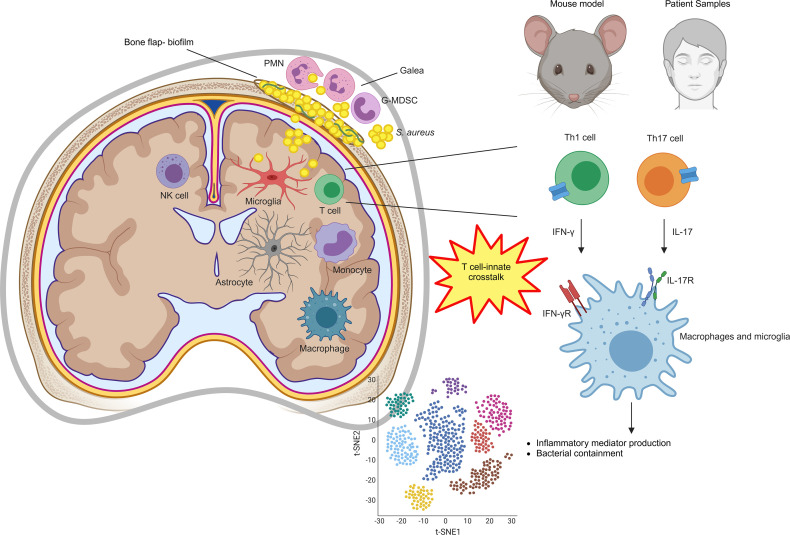

## Introduction

Craniotomy is performed to access the brain for the treatment of numerous neurological conditions including tumors, epilepsy, and aneurysm (1). During craniotomy, a skull fragment (bone flap) is removed and replaced intraoperatively. Despite prophylaxis and sterile surgical practices, approximately 1%–5% of craniotomies are complicated by infection, half of which are attributed to *Staphylococcus aureus* that forms a biofilm on the bone flap (2–4). These infections pose severe health risks, require rehospitalizations, and elevate healthcare costs (5, 6), where chronic infection is driven by the antiinflammatory and antibiotic tolerance properties of biofilm (7). Therefore, timely management of craniotomy infections is crucial and requires a rigorous treatment regimen involving wound debridement, prolonged antibiotic administration, and subsequent cranioplasty if the infected bone flap is not salvageable (8, 9). This highlights the need to better understand the host-pathogen dynamic during craniotomy infection to advance the possibility of immune-targeted therapies to mitigate biofilm growth in combination with antibiotics.

Our laboratory has developed a mouse model of *S*. *aureus* craniotomy infection to study host-pathogen crosstalk (10–13). This model achieves bona fide biofilm formation in terms of antibiotic tolerance, chronicity, and structure that displays good fidelity to human craniotomy infection (4, 10, 14). We previously identified a compartmentalized immune response during craniotomy infection, with differential leukocyte influx observed in distinct tissue niches (10, 15). Of interest was preferential T cell recruitment to the brain, which was largely absent on the bone flap (biofilm nidus) or s.c. galea (12); however, the functional importance of T cells during *S*. *aureus* craniotomy infection is unknown. In the current study, a critical role for adaptive immunity in bacterial containment was observed using *Rag1*-deficient (*Rag1*^–/–^) animals, which displayed significantly increased bacterial burdens. This was attributed to CD4^+^ T cells since CD4^+^ T cell depletion in WT mice also resulted in *S*. *aureus* outgrowth. Brain CD4^+^ T cell infiltrates were characterized by a robust IFN-γ and IL-17 signature that was independently validated by scRNA-Seq, as revealed by enriched *Tbx21* and *Cxcr3* versus *Rorc* and *Ccr6* expression, respectively. Adoptive transfer studies in *Rag1*^–/–^ animals established that both Th1 and Th17 cells were important for preventing bacterial outgrowth, with a critical window identified during acute infection. scRNA-Seq analysis of CD45^+^ cells recovered from the brains of infected WT and *Rag1*^–/–^ mice revealed a robust IFN-γ–dependent transcriptional signature in microglia, macrophages, and granulocytes. CellPhoneDB (16) identified strong interactions between T cells and monocytes/macrophages in the mouse model that was also observed in scRNA-Seq datasets from patients with craniotomy infection. The importance of proinflammatory Th cell responses was demonstrated when IL-17A activity was blocked in *Ifng*^–/–^ mice, which recapitulated the defects in bacterial containment observed in *Rag1*^–/–^ animals. Collectively, these findings reveal a critical role for IFN-γ– and IL-17A–producing CD4^+^ T cells in bacterial containment during *S*. *aureus* craniotomy infection mediated by effects on innate immune cell activation, underscoring the importance T cell–innate immune crosstalk during *S*. *aureus* biofilm infection.

## Results

### CD4^+^ T cells are critical for bacterial containment during S. aureus craniotomy infection.

**Figure 1:**
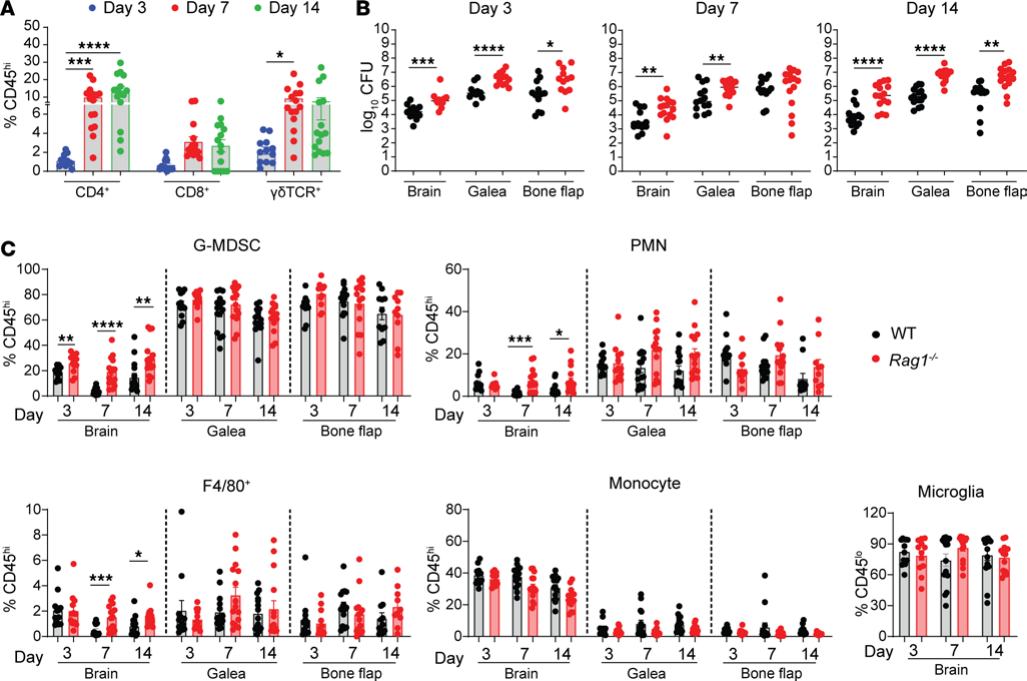
Importance of adaptive immunity during craniotomy infection. (**A**) WT mice were subjected to *S*. *aureus* craniotomy infection, whereupon brain tissues were collected at the indicated intervals to quantify various T cell populations by flow cytometry (*n* = 12–15 biological replicates combined from 3 independent experiments). (**B**) Bacterial burdens were quantified from the brain, galea, and bone flap of WT and *Rag1^–/–^* animals (*n* = 12–15/group) at the indicated intervals combined from 3 independent experiments. (**C**) Flow cytometry was performed to quantify G-MDSCs (CD11b^hi^Ly6C^+^Ly6G^+^F/480^–^), PMNs (CD11b^lo^Ly6C^+^Ly6G^+^F/480^–^), F4/80^+^ cells (CD11b^lo^Ly6C^+^Ly6G^+^F/480^+^), and monocytes (Ly6C^+^Ly6G^–^) in the brain, galea, and bone flap as well as microglia (CD45^lo^CX3CR1^+^) in the brain of WT and *Rag1^–/–^* mice at days 3, 7, and 14 after infection. Results are reported as the percentage of live CD45^+^ cells combined from 3 independent experiments (*n* = 12–15 mice/group) and represent mean ± SEM. *P < 0.05; **P < 0.01; ***P < 0.001; ****P < 0.0001. Two-way ANOVA with Tukey’s correction (**A**) or unpaired 2-tailed Student’s *t* test were used (**B** and **C**).

Although our prior reports revealed the preferential recruitment of lymphocytes to the brain during craniotomy infection (12, 13), their molecular characteristics and functional relevance remained unknown. We first assessed the frequency and kinetics of T cell infiltration in the brain, which revealed a time-dependent increase in CD4^+^ and γδ T cells (Figure 1A). The importance of adaptive immunity was demonstrated by elevated bacterial burdens in *Rag1*^–/–^ mice in the brain, s.c. galea, and bone flap with the most dramatic changes evident at day 14 after infection (Figure 1B). This coincided with significant increases in granulocytes (polymorphonuclear neutrophils [PMNs] and granulocytic myeloid-derived suppressor cells [G-MDSCs]) and Ly6G^+^Ly6C^+^F4/80^+^ cells in the brains of *Rag1*^–/–^ animals (Figure 1C). Similar trends were observed for absolute cell counts in the brain (Supplemental Figure 1A; supplemental material available online with this article; https://doi.org/10.1172/jci.insight.183327DS1).

**Figure 2:**
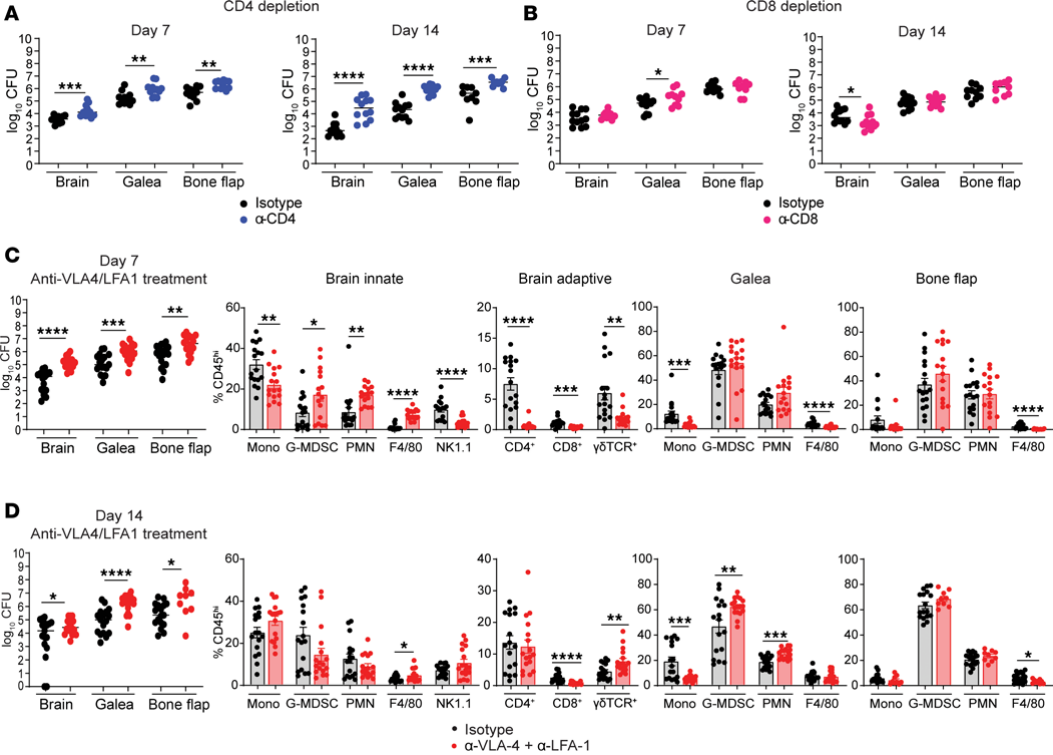
CD4^+^ T cells are critical for controlling craniotomy infection. (**A** and **B**) Bacterial burdens in WT mice following CD4 (**A**) or CD8 (**B**) depletion. Results are combined from 2 independent experiments (*n* = 10–12 mice/group). (**C** and **D**) WT animals received VLA-4 and LFA-1 or isotype-matched control antibodies to quantify bacterial burdens and immune cell infiltrates in various tissues at days 7 (**C**) or 14 (**D**) after infection. Results represent mean ± SEM combined from 3 independent experiments (*n* = 16–17/group). *P < 0.05; **P < 0.01; ***P < 0.001; ****P < 0.0001; unpaired 2-tailed Student’s *t* test.

We next depleted major T cell populations in WT mice to assess their role during craniotomy infection. CD4^+^ T cells were critical for infection containment as anti-CD4 treatment significantly increased bacterial burdens across all tissues examined (Figure 2A). In contrast, CD8^+^ T cell depletion only enhanced *S*. *aureus* titers in the galea at day 7 (Figure 2B), supporting the importance of CD4^+^ T cells for infection containment. G-MDSC and PMN infiltrates were significantly elevated following CD4^+^ T cell depletion at day 14 after infection (Supplemental Figure 1B), which recapitulated phenotypes in *Rag1*^–/–^ mice (Figure 1C). In contrast, granulocyte influx remained largely unchanged in all tissues following CD8 depletion, whereas minor changes in monocyte recruitment were observed in the brain at day 14 after infection (Supplemental Figure 1C), presumably due to reduced bacterial burden at this interval (Figure 2B). Since a sizable population of γδ T cells was recruited to the brain we examined their functional importance using a depletion strategy, which resulted in a modest increase in bacterial burdens but minimal effect on leukocyte infiltrates (Supplemental Figure 2). B cell recruitment to the brain was limited during *S*. *aureus* craniotomy infection (Supplemental Figure 3C), which was supported by our prior single-cell RNA-Seq (scRNA-Seq) studies (12).

As an independent strategy to demonstrate the importance of T cells during *S*. *aureus* craniotomy infection, WT mice received antibodies against VLA-4 (α_4_β_1_) and LFA-1 (CD11a/CD18) (17). Blocking both integrins significantly reduced CD4^+^ and CD8^+^ T cell recruitment to the brain at day 7 after infection, resulting in higher infectious burdens compared with isotype-treated mice concomitant with increased G-MDSC and PMN influx (Figure 2C), resembling phenotypes in *Rag1*^–/–^ animals (Figure 1C). Absolute cell counts revealed similar trends in the brain at day 7 following VLA-4/LFA-1 blockade (Supplemental Figure 1D). Surprisingly, anti–VLA-4/LFA-1 failed to prevent CD4^+^ T cell migration into the brain at day 14 after infection, despite persistent changes in bacterial burdens and innate immune cell infiltrates (Figure 2D), revealing a sustained footprint with early T cell blockade. This may be explained by alternative routes of T cell entry into the CNS (18, 19), development of VLA-4/LFA-1 neutralizing antibodies over time, and/or expansion of the small number of T cells that reached the brain in the face of integrin blockade. Indeed, CD4^+^ T cells infiltrating the brain were characterized by increased CD69, CD25, CD44, and Ki67 expression, reflective of an activated, proliferative state, compared with the blood, where T cells displayed a resting phenotype (Supplemental Figure 3, A and B). Collectively, these data demonstrate that CD4^+^ T cells are important for preventing bacterial outgrowth during *S*. *aureus* craniotomy infection.

### Temporal and transcriptional heterogeneity of T cell infiltrates during S. aureus craniotomy infection.

**Figure 3:**
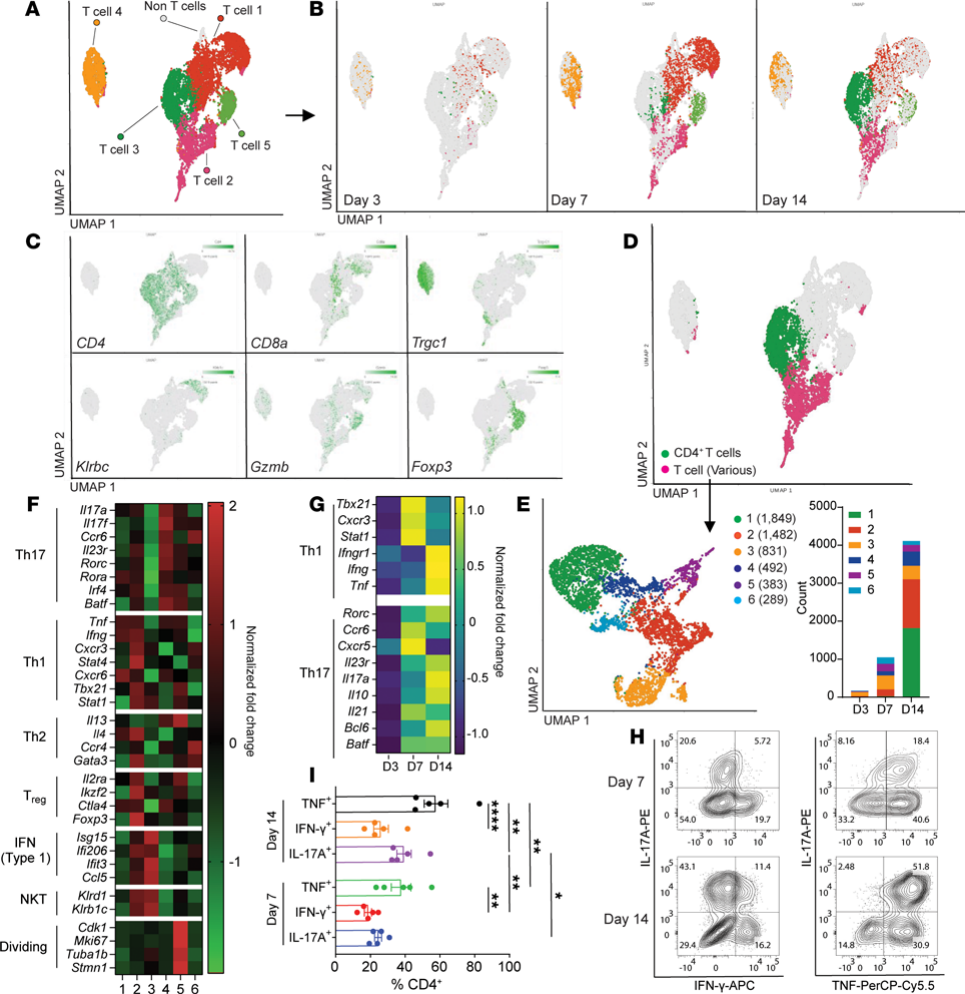
Single-cell transcriptomics reveals heterogeneity in brain CD3^+^ infiltrates during craniotomy infection. CD3^+^ cells were enriched from the brains of WT mice at days 3, 7, and 14 following craniotomy infection for scRNA-Seq. (**A** and **B**) Integrated UMAP representation of lymphoid cells and corresponding cluster identities separated according to time point. (**C**) Canonical genes associated with various T cell populations projected onto the UMAP. (**D** and **E**) CD4^+^ cells were integrated and reclustered to generate a new aggregated UMAP with the relative abundance of each cluster as counts. (**F**) Heatmap depicting the identity of various T cell clusters identified in **E** based on relative gene expression of prototypical genes for various T cell populations. (**G**) Heatmap reporting the temporal expression of canonical Th1- and Th17-associated signatures in the CD4^+^ cluster in **D**. (**H** and **I**) Quantification of IFN-γ, IL-17, and TNF expression in CD4^+^ T cells from the brains of WT mice ex vivo at days 7 and 14 after infection. Each data point in **I** represents CD4^+^ T cells pooled from 5 mice/group compiled from 5 independent experiments. *P < 0.05; **P < 0.01; ****P < 0.0001; 2-way ANOVA with Tukey’s correction.

To examine the transcriptional diversity of T cells and how this evolves during craniotomy infection, scRNA-Seq was performed on CD3^+^ cells recovered from the brains of WT mice at days 3, 7, and 14 after infection. A total of 27,209 high-quality transcriptomes were obtained and cluster identities were established by applying a Uniform Manifold Approximation and Projection (UMAP) clustering algorithm followed by data integration into a single sample, whereupon the Mouse Primary Cell Atlas (20) was used for cluster identification that revealed 5 distinct T cell clusters (Figure 3A). Clusters 1–3 possessed a CD4 signature and represented a major fraction of CD3^+^ cells at days 7 and 14 after infection (Figure 3, B and C), validating our flow cytometry findings (Figure 1A). Some myeloid populations were also present (Supplemental Figure 4) but were not included in the analysis to focus on T cell transcriptional profiles.

Expression of lineage markers revealed the presence of CD4^+^ (clusters 1, 2, 3, and 5), γδ (cluster 4), and CD8^+^ T cells (cluster 1) (Figure 3, A and C). Interestingly, Foxp3^+^ Tregs were enriched in cluster 5 and displayed a strong granzyme B signature (Figure 3C) reminiscent of noncanonical Tregs that inhibit T cell activation via their cytolytic activity in response to *S*. *aureus* (21). However, a sizable Foxp3^+^ T cell population was not detected in the infected brain by flow cytometry, and Treg depletion using anti-CD25 had no effect on bacterial burden (Supplemental Figure 5), suggesting that Tregs do not significantly influence craniotomy infection outcome. A strong proinflammatory signature was associated with brain γδ T cells (Supplemental Figure 6) that displayed an activated phenotype (Supplemental Figure 6E); however, their importance is unclear since only minor increases in *S*. *aureus* burden were observed following γδ T cell depletion (Supplemental Figure 2).

To gain additional insights into the transcriptional attributes of CD4^+^ T cells, clusters 2 and 3, which expressed the highest levels of CD4 transcripts, were reclustered (Figure 3D). This resulted in 6 distinct populations with an expansion of clusters 1, 2, and 4 at day 14 after infection (Figure 3E). Cluster 2 was associated with a Th1-like signature (*Ifng*, *Tnf*, *Cxcr3*, *Stat1*, *Tbx21*) along with some Treg genes (*Il2ra*, *Ikzf2*, *Foxp3*) (Figure 3F). The importance of Treg gene expression in a Th1-like population is unclear but could reflect plasticity since a small number of Tregs can lose FoxP3 expression and acquire T-bet^+^ Th1 phenotypes under certain inflammatory conditions (22, 23). In contrast, cluster 4 exhibited a Th17-like signature (*Il17a*, *Il17f*, *Ccr6*, *Il23r*, *Rorc, Rora*) (Figure 3F). Th1 and Th17 transcriptional profiles were more pronounced over time (Figure 3G), which was validated by robust IFN-γ, IL-17, and TNF production by brain CD4^+^ T cell infiltrates at these intervals (Figure 3, H and I). Cluster 1 was more heterogenous and lacked a distinct transcriptional profile, whereas Type I IFN (*Isg15*, *Ifi206*, *Ifit3*, *Ccl5*) and proliferative signatures (*Cdk1*, *Mki67*, *Tuba1b*) were observed in clusters 3 and 5, respectively (Figure 3F), whose numbers remained stable between days 7 and 14 after infection (Figure 3E). Collectively, these findings demonstrate that brain CD4^+^ infiltrates during craniotomy infection are transcriptionally heterogenous and associated with prominent Th1- and Th17-like signatures.

### Th1 and Th17 cells are critical for bacterial containment during S. aureus craniotomy infection.

**Figure 4:**
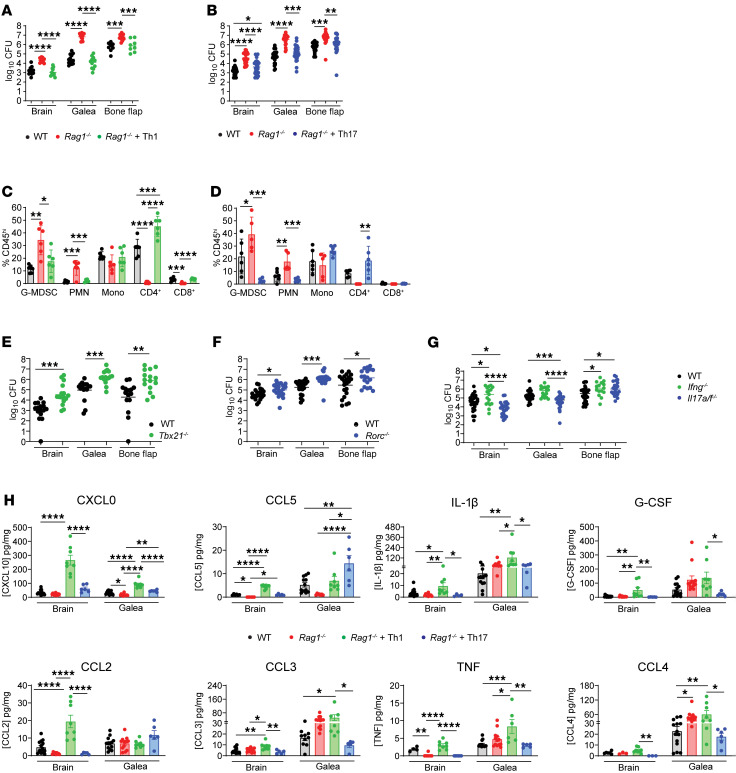
Th1 and Th17 cells are important for bacterial containment during *S*. *aureus* craniotomy infection. (**A**–**D**) Naive CD4^+^ cells were skewed to a Th1 or Th17 phenotype in vitro and adoptively transferred into *Rag1^–/–^* mice at day –1 prior to *S*. *aureus* craniotomy infection, whereupon bacterial burden (combined from 2–3 independent experiments (*n* = 12–26/group) (**A** and **B**) and immune cell infiltrates in the brain (*n* = 5/group; 1 example representative of 2–3 independent experiments) (**C** and **D**) at day 14 were quantified. (**E** and **F**) Bacterial abundance was assessed in the brain, galea, and bone flap of WT and *Tbx21*^–***/***–^ (**E**) or *Rorc^–/–^* (**F**) animals at day 14 after infection. Results are combined from 3–4 independent experiments (*n* = 16–22 mice/group). (**G**) Bacterial burdens were assessed in the brain, galea, and bone flap of *Ifng^–/–^* and *Il17a/f^–/–^* mice at day 14 after infection. Results combined from 3–4 independent experiments (*n* = 17–28 mice/group). (**H**) Cell-free homogenates from the brain and galea of WT, *Rag1*^–/–^, and *Rag1^–/–^* mice receiving Th1 or Th17 adoptive transfer were quantified for inflammatory mediator expression at day 14 after infection (*n* = 4–14/group). **A**, **B**, **E**, **F**, **G**, and **H** represent mean ± SEM, and **C** and **D** reflect mean ± SD. **A**–**D**, **G**, and **H** used 1-way ANOVA with Tukey’s correction, and **E** and **F** used unpaired 2-tailed Student’s *t* test. *P < 0.05; **P < 0.01; ***P < 0.001; ****P < 0.0001.

Although our flow cytometry findings and scRNA-Seq identified prominent Th1 and Th17 infiltrates during craniotomy infection, the functional importance of either subset on disease outcome was unknown. Adoptive transfer of in vitro polarized Th1 or Th17 cells returned the elevated bacterial burdens in the brain, galea, and bone flap of *Rag1^–/–^* mice to that of WT animals (Figure 4, A and B), suggesting a critical role for both Th subsets in preventing *S*. *aureus* outgrowth. Th1 and Th17 transfer also reduced the frequency of G-MDSC and PMN infiltrates in the brain of *Rag1^–/–^* recipients compared with *Rag1^–/–^* mice alone (Figure 4, C and D), suggesting that CD4^+^ T cells regulate granulocyte recruitment during craniotomy infection. Similar trends were observed with absolute cell counts but were less pronounced due to variability between individual animals (Supplemental Figure 7A). The importance of each Th subset was validated in *Tbx21^–/–^* and *Rorc^–/–^* mice (24) where bacterial abundance was significantly higher in both strains at day 14 after infection (Figure 4, E and F), although leukocyte recruitment was largely unaffected (Supplemental Figure 7, B and C). Collectively, these data highlight the importance of Th1 and Th17 responses in bacterial containment during craniotomy infection.

Interestingly, CD4^+^ cells recovered from the brains of Th17 recipient *Rag1^–/–^* mice revealed a strong IFN-γ signature despite robust IL-17A and no IFN-γ production prior to adoptive transfer (Supplemental Figure 8A). This led us to compare cytokine signatures of CD4^+^ T cells recovered from the brain and blood of WT and *Rag1^–/–^* animals following Th17 transfer. Cells in the blood remained largely quiescent; however, CD4^+^ T cells recovered from the brains of Th17 recipient *Rag1^–/–^* mice again produced higher levels of IFN-γ (Supplemental Figure 8B). To further explore the functional importance of IFN-γ and IL-17 in regulating craniotomy infection outcome, we next examined *S*. *aureus* abundance in *Ifng^–/–^* and *Il17a/f^–/–^* mice. Bacterial burdens were significantly increased in the brain and bone flap of *Ifng^–/–^* mice compared with WT animals at day 14 after infection, whereas unexpectedly, *S*. *aureus* abundance was reduced in the brain and galea of *Il17a/f^–/–^* mice but elevated in the bone flap compared with WT animals (Figure 4G).

Based on the critical role of CD4^+^ T cells in preventing *S*. *aureus* outgrowth in vivo, we next focused on how T cells shape innate immune cell responses during craniotomy infection. This is because Th1 and Th17 cells do not exhibit direct antimicrobial activity and likely exert their beneficial effects by programming leukocyte activation. IL-10 production was significantly increased in Ly6G^+^Ly6C^+^ granulocytes infiltrating the brain of *Rag1^–/–^* animals, and it was reversed following Th1 but not Th17 adoptive transfer (Supplemental Figure 8C), which is likely explained by the negative regulation of IL-10 by IFN-γ (25, 26). Furthermore, MHC class II expression was significantly reduced in monocytes and microglia in the brain of *Rag1^–/–^* mice that returned at or near WT levels following Th1 or Th17 adoptive transfer, which supports Th-dependent MHC class II regulation (Supplemental Figure 8C). Since *Rag1^–/–^* animals exhibited alterations in leukocyte recruitment and activation, we next examined whether this coincided with changes in the inflammatory milieu. Several cytokines (TNF, IL-1β) and chemokines (CXCL10, CCL2, CCL5) were reduced in the brains of *Rag1^–/–^* animals at day 14 after infection, which were restored or exceeded WT levels mainly with Th1 adoptive transfer, whereas Th17 cells were less effective (Figure 4H). The exaggerated production of mediators following Th1 adoptive transfer in *Rag1^–/–^* recipients compared with WT animals was restricted to chemokines with known IFN-γ dependence (CXCL10) (27) or described as Th1-chemokines (CCL2 and CCL5) (28). Fewer changes in cytokine/chemokine expression were detected in the galea of WT versus *Rag1^–/–^* mice, although some movement was observed following Th1 or Th17 transfer (Figure 4H). Collectively, these data indicate that proinflammatory Th1 and Th17 responses augment innate immune cell activation during *S*. *aureus* craniotomy infection.

To determine whether the ability of Th1 and Th17 cells to prevent *S*. *aureus* outgrowth in *Rag1^–/–^* mice was dependent on prior skewing to these phenotypes, we next examined the effect of nonpolarized bulk CD4^+^ T cells or Th0 cells. Interestingly, both populations significantly lowered *S*. *aureus* burdens compared with *Rag1^–/–^* mice, suggesting that CD4^+^ cells are capable of mediating protection in vivo regardless of their initial phenotype (Supplemental Figure 9, A and B). Importantly, both bulk and Th0 CD4^+^ T cell populations displayed a strong IFN-γ signature in vivo (Supplemental Figure 9C), demonstrating preferential Th1 skewing during *S*. *aureus* craniotomy infection in agreement with our previous findings (Supplemental Figure 8, A and B).

**Figure 5:**
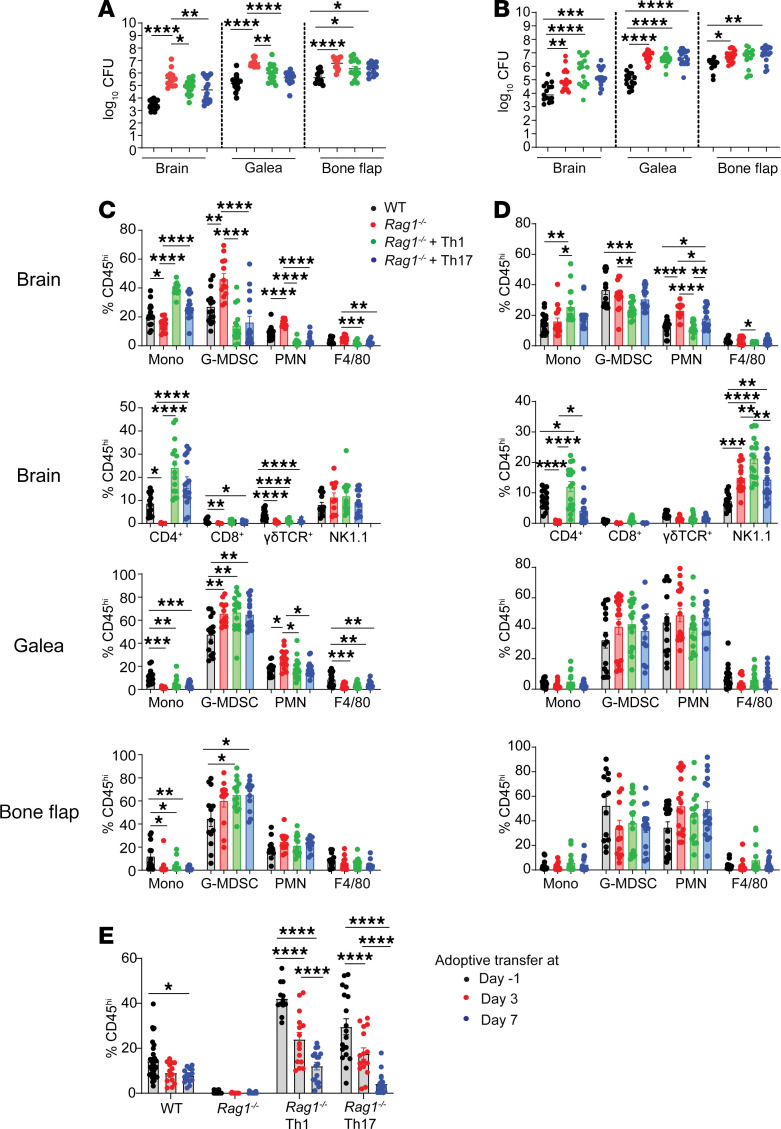
Th1 and Th17 cells are important during acute craniotomy infection to prevent *S*. *aureus* outgrowth. (**A**–**D**) Naive CD4^+^ cells were skewed to a Th1 or Th17 phenotype in vitro and adoptively transferred into *Rag1^–/–^* mice at day 3 (**A** and **B**) or 7 (**C** and **D**) after infection, whereupon bacterial burden (**A** and **B**) and leukocyte infiltrates (**C** and **D**) in the brain, galea, and bone flap were quantified at day 14 after infection (*n* = 13–18/group). (**E**) Percentage of brain CD4^+^ cells in each of the adoptive transfer window paradigms at day 14 after infection. Data in **E** were regraphed from Figure 4, C and D, (for day –1) and from **C** and **D** (for days 3 and 7) to highlight the progressive decrease in T cell recruitment over time. Results are combined from 3–4 independent experiments presented as mean ± SEM. One-way ANOVA (**A**–**D**) and 2-way ANOVA (**E**) with Tukey’s correction were used. *P < 0.05; **P < 0.01; ***P < 0.001; ****P < 0.0001.

The induction of antigen-specific (Ag-specific) T cell responses typically occurs within weeks after Ag exposure in naive individuals; however, *S*. *aureus* produces superantigens (SAgs) that can activate T cells in an Ag-independent manner (29, 30). To explore the protective window of T cell action during craniotomy infection, adoptive transfer was delayed to either days 3 or 7 after infection. Although bacterial burdens were significantly reduced in *Rag1^–/–^* mice receiving Th1 or Th17 cells at day 3 (Figure 5A), delaying adoptive transfer until day 7 after infection failed to prevent *S*. *aureus* outgrowth (Figure 5B). Similar findings were observed with leukocyte infiltrates, where monocyte, G-MDSC, and PMN recruitment to the brain was restored to WT levels when *Rag1^–/–^* mice received Th1 or Th17 cells at day 3 (Figure 5C) but were less effective when adoptive transfer was initiated at day 7 after infection (Figure 5D). Interestingly, T cell recruitment to the brains of *Rag1^–/–^* mice varied depending on the time of adoptive transfer, with maximal CD4^+^ influx seen at day –1, that progressively declined as transfer was delayed (Figure 5E), coinciding with the ability to control bacterial outgrowth (Figure 4, A and B). These findings establish a critical window for T cell activity during acute craniotomy infection, highlighting the importance of T cells in shaping the immune landscape to promote bacterial containment.

### Single-cell transcriptomics identifies a prominent IFN-γ signature in innate immune cells during craniotomy infection.

**Figure 6:**
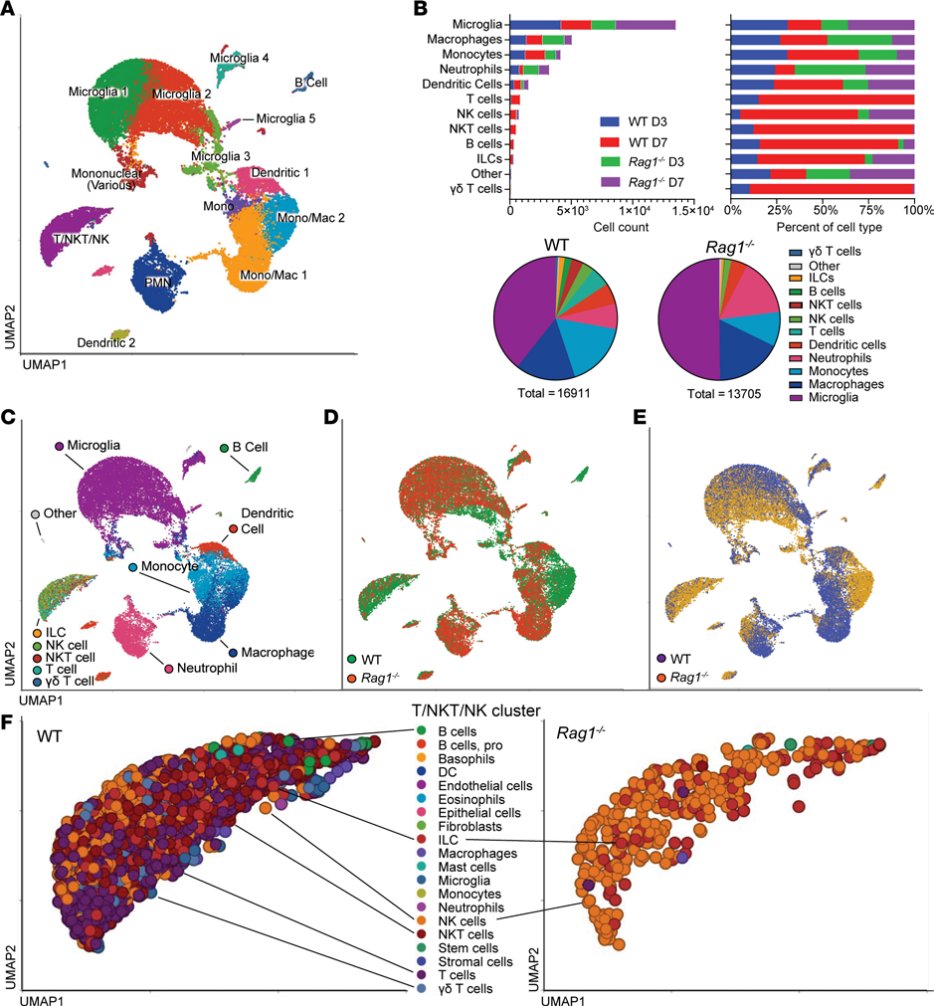
Single-cell transcriptomics of WT versus *Rag1^–/–^* immune populations during craniotomy infection. Viable CD45^+^ leukocytes were recovered from the brains of WT and *Rag1^–/–^* mice (*n* = 10/group) at days 3 and 7 after infection for scRNA-Seq. (**A** and **B**) Integrated UMAP representation of 30,616 sequenced cells (**A**) and corresponding cluster identities (**B**) summarized in aggregate by cell type and relative abundance. (**C**–**E**) UMAP (**C**) of collapsed immune populations separated by genotype (**D**) and time point (**E**). (**F**) Demonstration of ILC and NK cells in the T/NKT/NK cluster in *Rag1^–/–^* mice.

**Figure 7:**
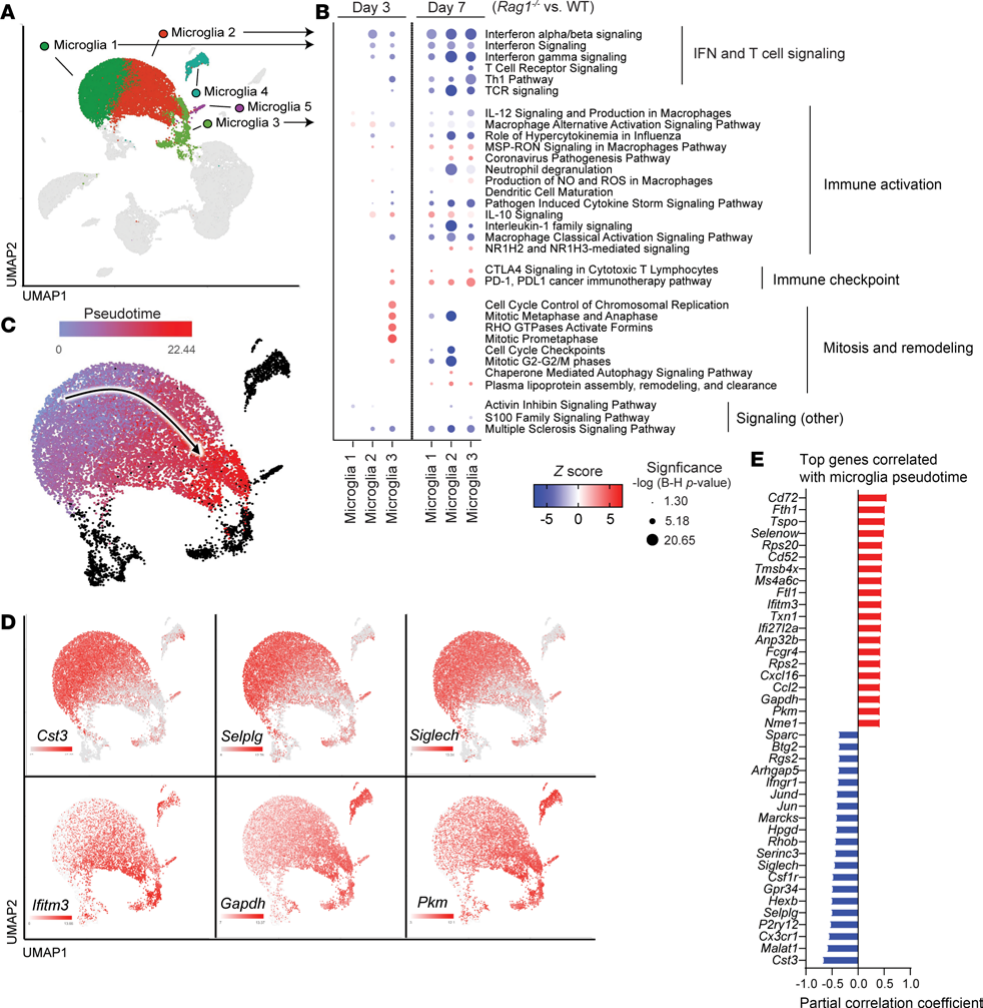
Microglia in *Rag1^–/–^* mice exhibit decreased proinflammatory activation during *S*. *aureus* craniotomy infection. Viable CD45^+^ cells were recovered from the brains of WT and *Rag1^–/–^* mice (*n* = 10/group) by FACS at days 3 and 7 following *S*. *aureus* craniotomy infection for scRNA-Seq. (**A** and **B**) UMAP reflecting microglial heterogeneity (**A**) and IPA (**B**) depicting the most significantly altered pathways between *Rag1^–/–^* and WT microglial clusters. (**C**–**E**) UMAP (**C**) with trajectory analysis of aggregated microglial clusters inferred by Monocle3 (arrow indicates predicted movement in pseudotime) with expression of select genes along the trajectory (**D**) and top genes correlated with pseudotime (**E**).

Given our findings where Th1 and Th17 cells influenced innate immune cell activation during craniotomy infection, scRNA-Seq was performed on CD45^+^ cells isolated from the brains of WT and *Rag1^–/–^* mice at days 3 and 7 after infection to obtain a more granular assessment of how innate responses are shaped in the presence or absence of lymphocyte subsets. Since T cell recruitment was restricted to the brain during craniotomy infection, we focused on transcriptional changes in this compartment. We first defined the aggregated immune cell landscape in the brains of WT and *Rag1^–/–^* mice, which revealed significant heterogeneity with 14 transcriptionally distinct clusters identified (Figure 6A), in agreement with previous findings (11, 12). Major cell populations included resident microglia, monocytes/macrophages, and granulocytes, with lymphoid and innate lymphoid subsets clustered into 1 group annotated as T/NK/NKT cells (Figure 6A). The remaining cells in the T/NKT/NK cluster in *Rag1^–/–^* mice represented NK and innate lymphoid cells (ILCs) (Figure 6F), validating their phenotype. A small B cell cluster was identified in WT mice (Figure 6, A and B), confirming the paucity of B cells detected by flow cytometry (Supplemental Figure 3C). To examine transcriptional profiles from a temporal perspective, clusters for each cell population were collapsed to create an aggregated UMAP (Figure 6C) that was separated based on mouse strain (Figure 6D) and time point (Figure 6E). For microglia, ingenuity pathway analysis (IPA) revealed significant reductions in Type I and Type II IFN signaling, T cell activation/signaling, and immune activation pathways including IL-12 and IL-1 across the top 3 microglial clusters in *Rag1^–/–^* mice primarily at day 7 after infection (Figure 7, A and B). Similar findings were observed with GSEA (Supplemental Figure 10A), underscoring the importance of IFN-regulated inflammatory responses in microglia during craniotomy infection. Interestingly, the PD-1/PD-L1 pathway was significantly increased in *Rag1^–/–^* microglial clusters at day 7 (Figure 7B), possibly reflecting an immunosuppressive or regulatory state. Trajectory analysis of the aggregated microglial clusters over pseudotime revealed a transition from a homeostatic (microglia 1) to proinflammatory (microglia 3) cluster (Figure 7C) based on canonical gene expression (Figure 7, D and E).

**Figure 8:**
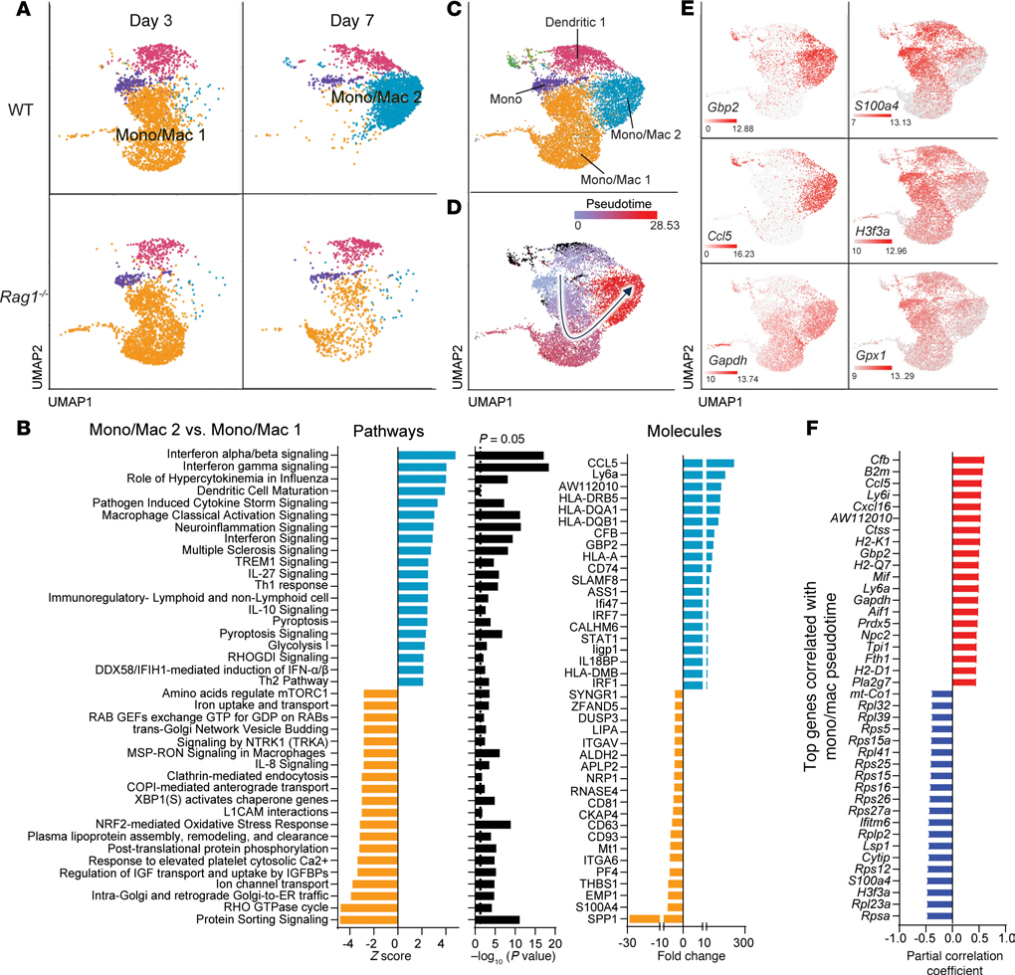
Monocyte/macrophage activation is regulated by adaptive immunity during *S*. *aureus* craniotomy infection. (**A**) UMAPs depicting the major monocyte/macrophage/DC clusters in the brains of WT versus *Rag1^–/–^* animals at days 3 and 7 following *S*. *aureus* craniotomy infection. (**B**) Top significantly expressed pathways for the unique Mono/Mac2 cluster versus Mono/Mac1 are presented along with predicted molecule expression. (**C** and **D**) UMAP clustering for pseudotime assessment (**C**) with trajectory analysis of monocyte/macrophage clusters inferred by Monocle3 (**D**), where the arrow depicts movement in pseudotime. (**E**) Expression of select genes along the trajectory. (**F**) Top genes correlated with pseudotime.

Examination of monocyte and macrophage transcriptional profiles revealed the appearance of a macrophage/monocyte cluster (Mono/Mac2) at day 7 after infection in WT animals that was largely absent in *Rag1^–/–^* mice (Figure 8A). This cluster was highly enriched in IFN and proinflammatory pathways compared with Mono/Mac1 (Figure 8B), including several IFN-γ–induced genes such as *Ccl5*, *Hladr*, *Hladq*, *Cd74*, *Stat1*, *Gpb2*, and *Hla1* (Figure 8B). In contrast, Mono/Mac1 was present in both WT and *Rag1^–/–^* mice at day 3 but was nearly absent in WT animals by day 7 after infection (Figure 8A). Unlike the IFN/proinflammatory phenotype of Mono/Mac2, the Mono/Mac1 cluster expressed pathways indicative of nutrient uptake/metabolism, reactive nitrogen/oxygen stress, and CXCL2 (IL-8 homolog) signaling (Figure 8B). Comparison of the Mono/Mac1 cluster between WT and *Rag1^–/–^* animals at day 3 after infection identified only 50 significantly differentially expressed genes (Supplemental Figure 11) representing 0.027% transcriptional coverage, which translated to a lack of significantly enriched pathways at this time point (Supplemental Figure 10B). This is in agreement with our finding that T cell infiltrates were less prominent at day 3 after infection compared with later time points (Figure 1A) and suggests the transition of Mono/Mac1 to Mono/Mac2 in WT animals at day 7 when robust T cell infiltrates are present. This was supported by UMAP reclustering for trajectory analysis (Figure 8C), which revealed a transition from Mono/Mac1 toward the more proinflammatory Mono/Mac2 cluster in pseudotime (Figure 8D) that was typified by increased expression of inflammatory and metabolic genes (Figure 8, E and F). In contrast, Mono/Mac1 exhibited increased levels of antiinflammatory (*S100a4*), antioxidant (*Gpx1*), and ribosomal subunit genes (Figure 8, E and F), in addition to increased IL-10 signaling at day 3 (Supplemental Figure 10C). Two DC clusters were also identified, although only 1 (Dendritic 1) had sufficient cell numbers for comparisons (Supplemental Figure 10B). Similar to microglia and monocytes/macrophages, the Dendritic 1 cluster in *Rag1^–/–^* mice had significant reductions in Type I and Type II IFN responses, IL-1 production, AIM2 inflammasome, and Ag presentation pathways primarily at day 7 after infection (Supplemental Figure 10, B and C).

Given our focus on the brain parenchyma since T cells selectively traffic to this compartment, only 1 PMN cluster was identified, which in *Rag1^–/–^* mice was associated with significant decreases in Type I and Type II IFN–related pathways and Ag processing and presentation that coincided with reduced expression of several IFN-induced molecules compared with PMNs from WT animals (Supplemental Figure 10D). However, PMNs infiltrating the brain of *Rag1^–/–^* mice exhibited a mix of proinflammatory (LCN2, APOE) and antiinflammatory (WFDC21, RETNLG) molecules that was most prominent at day 7 after infection along with enhanced PD-1/PD-L1 pathway expression (Supplemental Figure 10D). Collectively, these findings reveal extensive innate-adaptive crosstalk in the brain during *S*. *aureus* craniotomy infection that is important for shaping the antibacterial response, as made evident by the dramatic reduction in IFN-γ signatures in monocytes/macrophages/microglia from *Rag1^–/–^* mice coincident with a failure in bacterial containment.

### Human and mouse T cells interact with monocytes/macrophages during craniotomy infection.

**Figure 9:**
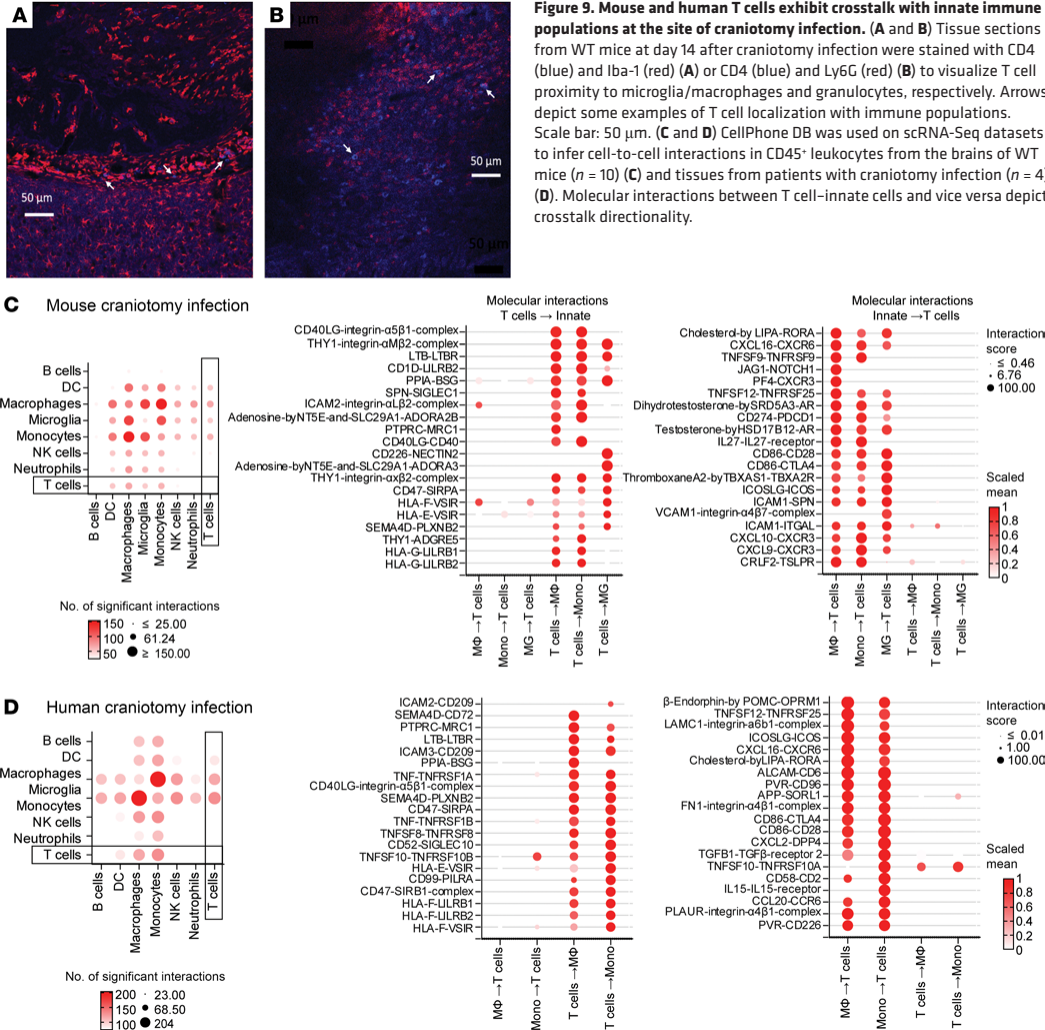
Mouse and human T cells exhibit crosstalk with innate immune populations at the site of craniotomy infection. (**A** and **B**) Tissue sections from WT mice at day 14 after craniotomy infection were stained with CD4 (blue) and Iba-1 (red) (**A**) or CD4 (blue) and Ly6G (red) (**B**) to visualize T cell proximity to microglia/macrophages and granulocytes, respectively. Arrows depict some examples of T cell localization with immune populations. Scale bar: 50 μm. (**C** and **D**) CellPhone DB was used on scRNA-Seq datasets to infer cell-to-cell interactions in CD45^+^ leukocytes from the brains of WT mice (*n* = 10) (**C**) and tissues from patients with craniotomy infection (*n* = 4) (**D**). Molecular interactions between T cell–innate cells and vice versa depict crosstalk directionality.

Since our scRNA-Seq studies suggested a significant degree of transcriptional crosstalk between infiltrating T cells and innate immune populations during *S*. *aureus* craniotomy infection, we next examined cellular proximity by immunofluorescence staining. Both microglia/macrophages and granulocytes were in close contact with CD4^+^ T cells as revealed by immunostaining with Iba-1 and Ly6G, respectively (Figure 9, A and B). To better appreciate the molecular interactions between T cells and their cognate innate partners, we leveraged the CellPhoneDB tool (16) using our scRNA-Seq datasets of CD45^+^ leukocytes recovered from infected WT mice and patients with craniotomy infection (4). In the mouse craniotomy infection model, T cells were found to primarily interact with monocytes and macrophages, with weaker associations with microglia and DCs (Figure 9C). Genes that were more highly expressed in mouse T cells that affected monocytes, macrophages, and microglia (T cells to innate cells) represented receptor-ligand interactions associated with T cell adhesion and activation (CD40L–integrin-α_5_β_1_, LTB-LTBR, SPN-SIGLEC1, ICAM2–integrin-α_L_β_2_, and CD40LG-CD40) but also molecules involved in inhibitory signaling and possible T cell exhaustion (CD1D-LILRB2, CD47-SIRPA, and HLA-G–LILRB1/LILRB2) (Figure 9C). Since interactions for a given cell type combination are not symmetrical, molecules that were more highly expressed by innate immune cells that affected T cell interactions were also identified. This again revealed a heightened T cell activation signature (IL-27–IL-27R, CD86-CD28, CD86-CTLA4, ICOSLG-ICOS, and CRLF2-TSLPR) and interactions for T cell chemotaxis, adhesion, and migration (CXCL16-CX3CR6, PF4/CXCR3, VCAM1–integrin-α_4_β_7_, ICAM1-ITGAL, CXCL10-CXCR3, and CXCL9-CXCR3) (Figure 9C).

T cell interactions with monocytes and macrophages were also prevalent in tissues from patients with craniotomy infection (Figure 9D), and several molecular interactions were similar to the mouse model, suggesting the importance of these networks. Molecules more highly expressed in human T cells that drove crosstalk with monocytes/macrophages included a large number of activating interactions (Sema4D-CD72, LTB-LTBR, TNF-TNFRSF1A/TNFRSF1B) that was also balanced by inhibitory associations (CD47-SIRPA, HLA-F–LILRB1/LILRB2, HLA-F–VSIR) as well as adhesion, chemotaxis, and migration (ICAM2/ICAM3-CD209, CD40L–integrin-α_5_β_1_, and CD99-PILRA) (Figure 9D). Genes enriched in human monocytes and macrophages that affected T cells during human craniotomy infection included those involved in activation (TNFSF12-TNFRSF25, ICOSLG-ICOS, CD86-CTLA4, and CD86-CD28) and adhesion and chemotaxis (LAMC1–integrin-α_6_β_1_, CXCL16-CXCR6, CXCL2-DPP4, CCL20-CCR6, and PLAUR–integrin α_4_β1 complex) (Figure 9D). The degree of concordance between T cell–monocyte/macrophage interactions in mouse and human craniotomy infection suggests the importance of these connections. To directly assess the consequence of innate–T cell crosstalk, mouse macrophages and microglia were exposed to live *S*. *aureus*, which elicited robust IFN-γ and IL-17A production from CD4^+^ T cells (Supplemental Figure 12, A and B). This was also seen in human monocytes, where coculture with autologous CD4^+^ T cells augmented monocyte bactericidal activity concomitant with increased IFN-γ production (Supplemental Figure 12, C and D). Collectively, these studies demonstrate that brain-infiltrating T cells are critical for inducing an IFN-dependent gene signature across all major innate immune cell populations during *S*. *aureus* craniotomy infection and form intricate associations with innate cells in both the mouse and human to modulate infection.

### IFN-γ and IL-17A cooperate to prevent S. aureus outgrowth during craniotomy infection.

**Figure 10:**
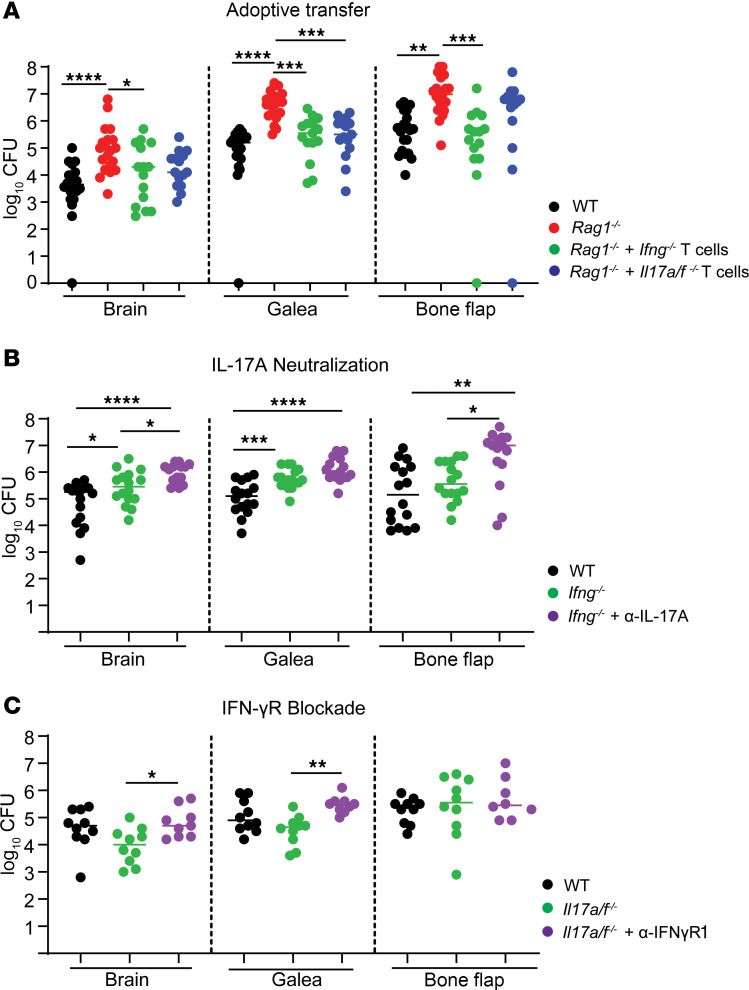
IFN-γ and IL-17A cooperate to prevent *S*. *aureus* outgrowth during craniotomy infection. (**A**) Nonpolarized CD4^+^ T cells from either *Ifng^–/–^* or *Il17a/f^–/–^* mice were adoptively transferred into *Rag1*^–/–^ animals at day –1 followed by quantification of bacterial abundance at day 14 after infection. Results combined from 3 independent experiments (*n* = 15–22 mice/group). (**B**) Bacterial burdens were assessed in the brain, galea, and bone flap of WT, *Ifng^–/–^*, or *Ifng^–/–^* mice receiving IL-17A neutralizing or isotype control antibodies (*n* = 16/group). Results combined from 3 independent experiments. (**C**) Bacterial burdens were assessed in the brain, galea, and bone flap of WT or *Il17a/f^–/–^* mice receiving IFN-γR1 blocking or isotype control antibodies (*n* = 9–10/group). Results were combined from 2–3 independent experiments; data are shown as mean ± SEM. *P < 0.05; **P < 0.01; ***P < 0.001; ****P < 0.0001; 1-way ANOVA with Tukey’s correction.

Our findings thus far support a role for both Th1 and Th17 cells in regulating innate immune activation and bacterial burden during *S*. *aureus* craniotomy infection. To directly assess the contribution of both IFN-γ and IL-17, nonpolarized CD4^+^ T cells from *Ifng^–/–^* or *Il17a/f^–/–^* mice were adoptively transferred into *Rag1^–/–^* animals. *Ifng^–/–^* T cells reduced infectious burdens in all compartments whereas *Il17a/f^–/–^* T cells were not as effective, only decreasing titers in the galea with a trending reduction in the brain (Figure 10A). This suggested the involvement of both IL-17A and IFN-γ in regulating infection. To test this possibility, *Ifng^–/–^* mice were treated with an IL-17A neutralizing antibody, which led to significant increases in bacterial titers in the brain, galea, and bone flap compared with WT and *Ifng^–/–^* mice alone, suggesting that both cytokines cooperate to limit bacterial outgrowth (Figure 10B). Conversely, IFN-γ receptor 1 (IFN-γR1; CD119) blockade in *Il17a/f^–/–^* mice exacerbated infectious burdens in the brain and galea compared with isotype-treated *Il17a/f^–/–^* animals (Figure 10C) confirming the cooperativity between the 2 cytokines and a dominant role for IFN-γ in infection containment. Collectively, these findings reveal that both Th1 and Th17 responses are critical for controlling *S*. *aureus* craniotomy infection. Nevertheless, T cell activity is insufficient for bacterial clearance under WT conditions since infection persists in the setting of an intact T cell response.

## Discussion

Here we established the functional importance of adaptive immunity during *S*. *aureus* craniotomy infection using *Rag1^–/–^* mice, which displayed higher infectious burdens compared with WT animals. CD4^+^ and γδ T cells represented the predominant T cell infiltrates in the brain with few CD8^+^ T cells or B cells, which was corroborated by our scRNA-Seq data. CD4^+^ T cells exhibited a strong IFN-γ and IL-17A signature, suggesting the involvement of Th1- and/or Th17-dependent proinflammatory responses. This was validated by the finding that adoptive transfer of in vitro–skewed Th1 or Th17 cells into *Rag1^–/–^* mice significantly decreased infectious burdens compared with *Rag1^–/–^* animals alone. Interestingly, adoptive transfer of nonskewed bulk CD4^+^ or Th0 T cells were also protective in *Rag1^–/–^* mice, and both acquired a robust IFN-γ signature in the brain, suggesting that CD4^+^ T cell populations can be shaped by the infection milieu to provide benefit. One caveat is that Th2 cell adoptive transfer was not assessed in this study since numerous attempts at Th2 polarization were unsuccessful. Th2 cells are less plastic, so they may have limited protection based on their inability to acquire IFN-γ production, although this remains speculative. Microglia and infiltrating monocytes/macrophages in the infected brain upregulated MHC class II expression that was significantly diminished in *Rag1^–/–^* mice, as revealed by both flow cytometry and scRNA-Seq. Class II levels were restored following T cell adoptive transfer into *Rag1^–/–^* animals, further establishing the need for an optimal IFN-γ response in regulating MHC class II expression and downstream proinflammatory effects. These findings corroborate previous studies where IFN-γ has been shown to mediate proinflammatory activity, in part, via MHC class II upregulation (31). Furthermore, several innate immune populations, not only in the brain but also the infected galea, exhibited a prominent IFN-γ gene signature (i.e., *H2Eb1*, *H2Ab1*, *H2Aa*, *Ccl5*, *Cd74*, *Stat1*) that was significantly reduced in analogous clusters from *Rag1^–/–^* mice. Surprisingly, despite this robust IFN-γ phenotype, the cytokine alone was dispensable, since adoptive transfer of *Ifng^–/–^* CD4^+^ T cells could still prevent *S*. *aureus* outgrowth in *Rag1^–/–^* mice, which was attributed to the action of IL-17 that was still produced by *Ifng^–/–^* CD4^+^ T cells. This was demonstrated by the finding that treatment of *Ifng^–/–^* mice with an IL-17A neutralizing Ab led to heightened bacterial burdens, recapitulating phenotypes seen in *Rag1^–/–^* animals. This work implicates the coordinated action of Th1 and Th17 cells in programming innate immunity during biofilm infection, corroborating prior studies and revealing an important role for Th1 and Th17 responses in mitigating *S*. *aureus* infection in various systemic and CNS disease models (32–36). Both Th1 and Th17 cells are critical for demyelination and motor pathology during experimental autoimmune encephalomyelitis (EAE), an animal model of multiple sclerosis (37). *Rag1^–/–^* mice did not display motor abnormalities following Th1 or Th17 adoptive transfer, suggesting that autoimmune-like pathology does not manifest during craniotomy infection during the timescale examined in this study.

The primary goal of scRNA-Seq was to characterize T cell heterogeneity and how this may shape innate immune cell activation. Our findings reveal the loss of a prominent proinflammatory macrophage/monocyte cluster in *Rag1^–/–^* animals concomitant with diminished proinflammatory signatures in additional innate cell populations, reflecting initial evidence of adaptive-innate immune crosstalk. We also leveraged the CellPhoneDB program to bioinformatically predict T cell interactions with various innate immune cell populations, an approach widely used in the field of transcriptomics (16). Extensive T cell–innate communication was observed in both patient samples and the mouse model at the site of craniotomy infection, reflecting a high degree of conservation across species. Several reciprocal activation signals were detected between T cells and monocytes/macrophages and microglia, in agreement with our findings during craniotomy infection where T cells displayed robust activation (CD44^+^, CD69^+^), proinflammatory (TNF^+^, IFN-γ^+^, and IL17A^+^), and proliferative (Ki67^+^) attributes. Of note, some inhibitory interactions were also observed that could reflect Treg crosstalk with their cognate innate partners; however, Treg depletion had no effect on craniotomy infection, making this possibility less likely. Although top interactions were not pursued in the current report, some findings validated our prior work, such as TNFR signaling, which was found to influence select macrophage and granulocyte responses to *S*. *aureus* (38), further supporting the existence of T cell–innate cell crosstalk. In addition, we demonstrated that macrophages/microglia as well as granulocytes are in close physical proximity to CD4^+^ T cells in vivo, reflecting their potential to interact. Finally, we also showed that T cell cytokine production was enhanced in the presence of both *S*. *aureus*–pulsed mouse and human APCs in vitro. Collectively, our results support a role for T cells in augmenting innate immune cell proinflammatory activity in response to *S*. *aureus* biofilm infection, supporting the crosstalk between these cell types. One surprising finding was that, despite a sizable γδ T cell infiltrate in the brain, these cells did not play a major role in controlling *S*. *aureus* craniotomy infection outcome, which differs from *S*. *aureus* skin and soft tissue infection or peritonitis where γδ T cells are protective (39–41). One possibility is that γδ T cells may receive inhibitory signals in the brain to limit their effector function, although this remains speculative.

The efficiency of effector CD4^+^ T cells is likely influenced by the nature and duration of *S*. *aureus* infection. For example, the protective window of CD4^+^ T cells in craniotomy infection was observed during acute intervals, and brain CD4^+^ T cell infiltrates were highly activated during the first 2 weeks of *S*. *aureus* craniotomy infection in WT animals based on characteristic surface marker and Ki67 expression. In contrast, other reports demonstrate that T cells were dispensable during early infection in a mouse model of *S*. *aureus* bacteremia and displayed characteristics of anergy during late-stage disease (42), and T cells recovered from animals during chronic *S*. *aureus* infection displayed diminished recall responses (43). It remains possible that CD4^+^ T cells may become anergic or exhausted beyond the 2-week interval examined in this study, which could contribute to the inability to achieve sterilizing immunity in craniotomy infection. Of note, G-MDSCs were a major infiltrate during craniotomy infection, and we and others have shown their importance in attenuating proinflammatory responses to *S*. *aureus* (12, 44–46). Therefore, chronic G-MDSC conditioning of the inflammatory milieu could be another mechanism to negate a protective role of T cells. Indeed, the existence of a G-MDSC/T cell axis can be inferred in our studies by the finding that G-MDSC infiltrates were expanded in the absence of T cells and returned to WT levels once T cell recruitment was restored. This is an interesting observation that might be linked to T cell mediators that influence G-MDSC antiinflammatory activity. However, this is highly speculative and warrants further experimentation to assess granulocyte function in the presence or absence of T cell infiltrates.

An open question is whether the CD4^+^ T cells that enter the brain during *S*. *aureus* craniotomy infection prevent bacterial outgrowth in an Ag-dependent or -independent manner. We are actively pursuing this issue, and both scenarios are not mutually exclusive. For example, the early window of CD4^+^ T cell protection that we identified following adoptive transfer in *Rag1^–/–^* mice (days –1 to 3 after infection) supports Ag-independent mechanisms given the timescale for induction of Ag-dependent responses. This is supported by the fact that *S*. *aureus* produces several SAgs that act as potent T cell mitogens (29, 30); therefore, the increased frequency of T cells observed in the brain at later intervals may stem from polyclonal expansion. On the other hand, the maximal window of T cell influx to the brain (i.e., days 7–14 after infection) would align with Ag-dependent T cell activation in draining cervical lymph nodes and subsequent migration to the brain. Indeed, the importance of T cell migration from the periphery was demonstrated using VLA-4 and LFA-1 blockade, which exacerbated bacterial burdens in WT mice; however, this finding is not sufficient to prove/disprove Ag specificity. A caveat with this approach is that T cells can utilize alternative adhesion molecules to extravasate (47); therefore, it is plausible that the residual T cells entering the brain following VLA-4/LFA-1 blockade could be expanded in an Ag-independent manner by *S*. *aureus* SAgs. Other cell types such as monocytes express VLA-4 and LFA-1 (48) and some alterations in innate immune cell recruitment were observed following VLA-4/LFA-1 inhibition, suggesting this may be a factor. CD4^+^ T cells were in close physical proximity to microglia/macrophages in the infected brain, which was validated bioinformatically using CellPhoneDB (16), supporting the possibility of Ag-dependent activation. Similarly, T cell–dependent induction of MHC class II on innate cell populations in the infected brain further supports the possible role of Ag-specific responses. Future studies incorporating spatial transcriptomics would provide unprecedented insights into potential molecular mechanisms of T cell–innate cell crosstalk during craniotomy infection.

Another important issue is how T cells respond to increasing bacterial burden, which may influence innate immune crosstalk. Our prior work in the craniotomy infection model has established that biofilm formation occurs at day 7 after infection since systemic antibiotics cannot clear *S*. *aureus* at this interval (14). By extension, this timing coincides with the window of T cell action reported in this study by adoptive transfer experiments. Specifically, introducing CD4^+^ T cells at days –1 or 3 after infection reduced bacterial burdens in *Rag1^–/–^* mice, whereas delaying T cell transfer until day 7, when a mature biofilm has formed, was not protective. This was also reflected by the finding that CD4^+^ T cell recruitment to the brains of *Rag1^–/–^* animals progressively decreased when adoptive transfer was delayed, with the most dramatic impairments observed at day 7 after infection, again aligning with the onset of biofilm development. Importantly, the latter paradigm still afforded 1 week for adoptively transferred T cells to reach the brain, a timescale sufficient for T cells to traffic to the infection site in WT animals, which does not further increase at day 14. Therefore, it is less likely that phenotypes would have emerged with the day 7 adoptive transfer paradigm at later intervals; nevertheless, this remains a caveat for consideration. Collectively, this suggests that CD4^+^ T cell recruitment and/or activation could be hampered by cues generated from infection milieu that evolve as bacteria transition from planktonic to biofilm growth, which remains to be determined.

There are several limitations of this study. First, there are recognized differences in the potency of select *S*. *aureus* virulence factors toward mouse versus human cells (49–51). Although our mouse model recapitulates many features of craniotomy infection in humans in terms of leukocyte infiltrates, MRI features, biofilm formation, inflammatory attributes (4, 10, 12), and the molecular interactions between T cells and innate immune cells identified in this study, differences in how species-restricted *S*. *aureus* factors differentially influence T cell responses in mice and humans cannot be ruled out. Second, the importance of IL-17F during craniotomy infection remains an open question since the cytokine was absent in *Il17a/f^–/–^* mice, whereas only IL-17A was neutralized in *Ifng^–/–^* animals. In addition, this work does not address the potential cooperation between γδ and CD4^+^ T cells in controlling bacterial outgrowth, which is plausible given the finding that γδ T cells are a major source of IL-17 during *S*. *aureus* infection in the periphery (41). Although B cell infiltrates are minimal during craniotomy infection, they may partially contribute to the phenotypes observed in *Rag1^–/–^* mice that can be explored in future studies with *muMT^–/–^* animals. Finally, the involvement of additional cytokines produced by activated CD4^+^ T cells, such as TNF and GM-CSF, in dictating craniotomy infection outcome remains a possibility and was not examined here.

Collectively, this work reveals a role for CD4^+^ T cells during *S*. *aureus* craniotomy infection by promoting innate immune cell proinflammatory activity and corroborates previous studies demonstrating the importance of T cell–mediated immune responses in the context of *S*. *aureus* infection in the CNS and periphery (33, 52–58). Of note, despite the presence of T cell infiltrates and their beneficial role in preventing *S*. *aureus* outgrowth during craniotomy infection as shown here, this is not sufficient to achieve sterilizing immunity, highlighting the complexity of biofilm infection and possible role of T cell anergy elicited by *S*. *aureus*-derived factors (42). Recent consensus statements have noted the challenges associated with treating *S*. *aureus* bone infections and placed a high priority on better understanding cellular immunity in this context (59, 60). However, it will be important to consider how the local tissue milieu affects immune programming given recent findings that granulocyte responses to *S*. *aureus* biofilm differ in distinct tissue niches (61). Gaining a deeper understanding of molecular mechanisms of T cell activity, exhaustion, specificity, and bacterial factors that regulate these processes will be necessary to develop more specific and translatable immune-directed therapies that can be combined with antibiotics to eradicate biofilm infections.

## Methods

### Sex as a biological variable.

Both male and female mice and human subjects were examined in this study, and similar findings are reported for both sexes.

### Mice.

*Rag1^–/–^* (RRID:IMSR_JAX:002216), *Il17a/f^–/–^* (RRID:IMSR_JAX:034140), *Ifng^–/–^* (RRID:IMSR_JAX:002287), *Tbx21^–/–^* (RRID:IMSR_JAX:004648), and *Rorc^–/–^* (RRID:IMSR_JAX:007571) mice were purchased from The Jackson Laboratory with age- and sex-matched C57BL/6J animals (RRID:IMSR_ JAX:000664) used as WT controls. Mice were housed in a BSL2 room in ventilated microisolator cages with a 12-hour light/dark cycle and ad libitum access to food and water.

### Mouse model of craniotomy infection.

*S*. *aureus* craniotomy infection was established in 8- to 12-week-old male and female mice as previously described with an inoculum of 1 × 10^3^ colony forming units (CFU) per bone flap (10, 11). Only infected animals were examined in this study due to paucity of T cells and other immune infiltrates in the brain and galea following sham craniotomy in the absence of infection (61).

### In vivo antibody-mediated approaches.

All antibodies for in vivo studies were purchased in a low-endotoxin, no-azide format from BioXCell (antibody information provided in Supplemental Table 1) and administered via i.p. injection. For each depletion/blocking strategy, mice received an equivalent amount of isotype-matched control Ab using the same dosing paradigm. For CD4^+^ and CD8^+^ T cell depletion, 300 μg of anti–mouse CD4 or anti–mouse CD8α was administered 3 days prior to craniotomy infection and every fourth day until sacrifice. For Treg depletion, animals received 400 μg of anti–mouse CD25 at day –1 and on the day of infection, with repeat injections every sixth day. For γδ T cell depletion, 300 μg of anti–mouse TCR γ/δ was given 2 days prior to infection and every 3 days until sacrifice. Cellular depletion was confirmed in all studies by evaluating target populations in the blood, spleen, and/or brain by flow cytometry. For VLA-4 and LFA-1 blockade, mice received 100 μg each of anti–mouse/human VLA-4 and anti–mouse LFA-1α 1 day prior to *S*. *aureus* infection and every other day until sacrifice. Effective blockade of T cell entry into the infected brain was confirmed by flow cytometry. For IL-17A neutralization in *Ifng^–/–^* mice, 1 mg of anti–mouse IL-17A was administered 1 day prior to *S*. *aureus* infection with repeat injections of 500 μg Ab every 2 days until sacrifice. For IFN-γR1 blockade, 300 μg of anti–mouse CD119 (IFN-γR1) was given 1 day prior to infection and every other day until sacrifice.

### In vitro CD4^+^ T cell polarization and adoptive transfer.

Naive CD4^+^ T cells (CD4^+^CD62^hi^CD44^lo^) were isolated from the spleens of 6- to 8-week-old mice using a MojoSort mouse naive T cell isolation kit (480040, BioLegend). T cells were cultured in RPMI supplemented with 10% FBS (HyClone-Cytiva), penicillin/streptomycin/fungizone (Thermo Fisher Scientific), 2 mM L-glutamine (HyClone-Cytiva), 1% HEPES (HyClone-Cytiva), and 0.01% β-mercaptoethanol (Thermo Fisher Scientific). For Th17 differentiation, naive T cells were seeded at a density of 1 × 10^6^/mL in a 96-well flat-bottom plate coated with anti-CD3ε (RRID: AB_11149115) and anti-CD28 (RRID: AB_11147170) and treated with 30 ng/mL IL-6 (catalog 575706), 10 ng/mL IL-23 (catalog 589004), 10 ng/mL IL-1β (catalog 575104), and 2 ng/mL TGF-β1 (catalog 781804) (all from BioLegend) along with 10 μg/mL each of anti–IFN-γ (RRID: AB_1089144), anti–IL-4 (RRID: AB_315316), and anti–IL-2 (RRID: AB_315292) for 3 days. For Th1 polarization, naive T cells were exposed to anti-CD3ε/anti-CD28 mouse T-activator Dynabeads (11456D, Thermo Fisher Scientific) at a 1:1 bead/cell ratio with 10 μg/mL anti–IL-4 (RRID: AB_315316), 10 ng/mL IL-2 (catalog 575406), and 10 ng/mL IL-12 (catalog 577004) (all from BioLegend) for 3 days.

On the day of adoptive transfer, Th1 and Th17 polarization efficiency was determined by intracellular staining with IFN-γ-APC (RRID:AB_315403) and IL-17A-PE (RRID:AB_315463) to ensure a total of 1 × 10^6^ Th1 or Th17 cells were delivered. For some experiments, 1 × 10^6^ Th0 (CD62^hi^CD44^lo^) or bulk CD4^+^ T cells were enriched from spleens of 6- to 8-week-old C57BL/6J mice using CD4 naive (catalog 480040) or total CD4 (catalog 480033) T cell MojoSort isolation kits (both from BioLegend), respectively, for adoptive transfer into *Rag1^–/–^* recipients. T cells were injected i.v. into recipient mice in 100 μL of 1× PBS via the retro-orbital sinus.

### Tissue processing and flow cytometry.

Tissues were recovered from mice to quantify bacterial burdens and immune cell populations in the brain, galea, and bone flap as previously described (11). For flow cytometry analysis, single-cell suspensions were incubated with Fc block (101320, BioLegend) for 10 minutes followed by staining with an antibody panel to quantify immune cell populations and T cell activation and proliferation (Supplemental Table 1).

For T cell intracellular staining, brain leukocytes were pooled to obtain sufficient numbers for analysis and enriched for CD3^+^ cells using a MojoSort Mouse CD3 Selection Kit (480031; BioLegend). Purified CD3^+^ cells were treated immediately ex vivo with a cocktail of PMA/ionomycin and brefeldin A (423304, BioLegend) for 4 hours. Next, cells were washed and incubated with Fc block (101320, BioLegend) for 10 minutes on ice prior to surface marker staining. Samples were then fixed and permeabilized using a Cyto-Fast Fix/Perm Buffer Set (426803, BioLegend) followed by intracellular cytokine staining (Supplemental Table 1). For detecting cytokine expression in innate immune cells, brain and galea samples from individual mice were incubated with brefeldin A immediately ex vivo for 4 hours and processed as described above. Dead cells for all flow cytometry analysis were excluded using a Zombie UV Fixable viability kit (423108, BioLegend). Samples were acquired on a LSRII Green cytometer and analyzed using FlowJo (RRID:SCR_008520) using the gating strategy presented in Supplemental Figure 13. For some experiments, counting beads (ACBP10010, Spherotech) were added after the final wash step before acquisition to determine absolute cell numbers in samples.

### Inflammatory mediator quantification.

Cytokine levels in cell-conditioned supernatants were quantified using mouse (catalog 560485) or human (catalog 560484) Th1/Th2/Th17 Cytometric Bead Arrays (both from BD Biosciences). Inflammatory mediator expression in cell-free tissue homogenates was quantified using Milliplex multianalyte bead arrays (catalog MCYTMAG70PMX25BK; MilliporeSigma). Values were normalized to total protein to correct for differences in tissue sampling size.

### Immunofluorescence staining.

Tissue sections encompassing the craniotomy infection site were processed for immunofluorescence staining as previously described (62). T cells were stained using biotin anti-mouse CD4 (RRID:AB_312710) and streptavidin-Brilliant Violet 421 (405226, BioLegend), microglia/macrophages with Iba-1 (RRID:AB_10583150) and donkey anti–rabbit IgG-Alexa Fluor 594 (RRID:AB_2340622), and granulocytes with anti–mouse Ly6G-Alexa Fluor 647 (RRID:AB_1134159). Sections were coverslipped in Prolong Anti-fade reagent prior to image acquisition by confocal microscopy (Zeiss LSM710) using a 20× oil objective.

### CD4^+^ T cell crosstalk with human monocytes and mouse macrophages and microglia in response to S. aureus.

Primary macrophages were generated from bone marrow isolated from the femurs and tibias of 8- to 12-week-old C57BL/6J mice (63), and primary microglia were prepared from the brains of 1- to 4-day-old C57BL/6J animals as previously described (11). Human PBMCs were obtained from the UNMC Elutriation Core, where CD4^+^ T cells were purified by FACS using human anti–CD3 Alexa Fluor 700 (RRID: AB_493740) and anti–CD4 PE (RRID: AB_2562053), and autologous monocytes were enriched using a MojoSort human pan monocyte isolation kit (480060, BioLegend). To assess how human CD4^+^ T cells affect monocyte bactericidal activity, gentamicin protection assays were performed as previously described (13). Briefly, human monocytes were incubated with live *S*. *aureus* at a multiplicity of infection (MOI) of 1:1 (bacteria/monocyte) for 1 hour, whereupon cells were treated with 100 μg/mL gentamicin to kill remaining extracellular bacteria. Next, autologous human CD4^+^ cells were added in the presence of 1 μg/mL gentamicin to prevent extracellular bacterial outgrowth and incubated for 24 hours, whereupon monocytes were lysed using sterile H_2_0 to quantify intracellular bacteria as a measure of bactericidal activity.

Conditioned medium was collected from mouse or human CD4^+^ T cells cocultured for 24 hours with *S*. *aureus* pulsed mouse macrophages and microglia or human monocytes, respectively, whereupon IFN-γ and IL-17 production was quantified using mouse (catalog 560485) or human (catalog 560484) Th1/Th2/Th17 cytometric bead arrays (both from BD Biosciences).

### scRNA-Seq and bioinformatics.

Two experimental paradigms leveraged scRNA-Seq in the mouse craniotomy infection model to define (a) T cell heterogeneity over the course of craniotomy infection with CD3^+^ cells enriched from the brains of WT animals collected at days 3, 7, and 14 after infection and (b) innate-adaptive immune crosstalk using CD45^+^ cells recovered from the brains of WT and *Rag^–/–^* mice at days 3 and 7 after craniotomy infection. For both studies target populations were enriched by FACS, whereupon scRNA-Seq was performed by the UNMC Genomics Core using the 10X Genomics platform as previously described (11, 12, 64). Briefly, cell viability, density, and debris were assessed using a Luna automated fluorescent cell counter (Logos Biosystems) before single-cell capture with a 10X Genomics instrument. Cells were then lysed, with RNA reverse transcribed and barcoded using a Chromium Single Cell 3′ Reagent Kit (v3.1; 10X Genomics) according to the manufacturer’s instructions. Illumina-compatible cDNA libraries were quantified with a Qubit-30 Fluorometer and assessed using a fragment analyzer before loading on a Novaseq6000 instrument at a final concentration of 300 pM for generation of 75 bp pair-end reads. Sequencing was performed to an average depth of 50,000–100,000 reads per cell. Sequencing data were aligned to the mouse genome using 10X Genomics Cell Ranger (RRID:SCR_016957) before importing into Partek Flow Genomics Suite (RRID: SCR_011860) for the remainder of the analysis. A standard quality control pipeline was implemented to safeguard against contamination by low-quality cells/reads. The resulting single-cell count matrices were normalized in Partek Flow using counts per million, add 1, and log base 2 transformed, and clustering was performed using the top 15 principal components. Cells were individually classified into cell types using SingleR (65) with the Immunological Genome Project Database (66) as a reference dataset. These assignments were used to classify graph-based clusters with their cellular identity. A Hurdle model was used to determine differential gene expression between clusters. Differential expression results were exported and further explored using IPA (RRID:SCR_008653).

Cell-to-cell interactions were predicted using the CellPhone DB program (16) in Partek Flow Genomics Suite on scRNA-Seq datasets from CD45^+^ cells in the brains of WT mice and tissues from patients with craniotomy infection. The human scRNA-Seq dataset representing 4 patients with craniotomy infection was previously generated by our laboratory and deposited in the GEO database (GSE249319) (4). Subject demographics and sample identity are described in Supplemental Table 2, where samples were collected intraoperatively from patients with craniotomy infection after informed consent as approved by the UNMC IRB (0241-18).

### Statistics.

Significant differences between groups were determined using either an unpaired 2-tailed Student’s *t* test, 1-way ANOVA, or 2-way ANOVA with Tukey’s multiple-comparison test using GraphPad Prism (RRID:SCR_002798). *P* < 0.05 was used to identify statistical significance for all analyses. For scRNA-Seq analysis, significant differences were determined using an FDR-adjusted *P* < 0.05. Figures were created using Partek Flow and GraphPad Prism.

### Study approval.

All animal experiments were approved by the UNMC IACUC (no. 16-123-10). Informed consent for procuring tissue samples from patients with craniotomy infection was obtained under a protocol approved by the UNMC IRB (no. 0241-18). Animal studies were conducted according to the recommendations in the *Guide for the Care and Use of Laboratory Animals* (National Academies Press, 2011) and comply with the Animal Research: Reporting of In Vivo Experiments guidelines (67).

### Data availability.

All supporting data values underlying the main and supplemental figures are provided in the Supporting Data Values file. The scRNA-Seq data are openly available in the GEO database (SuperSeries GSE264738 and GSE249319).

## Author contributions

GK, ZVR, LEK, and TK designed experiments. GK, ZVR, and RWF conducted experiments. GK wrote the manuscript. All authors edited and approved the final manuscript.

## Supplementary Material

Supplemental data

Supporting data values

## Version 1 02/24/2025

Electronic publication
