# Supplementary material for: CD4+ T cell–innate immune crosstalk is critical during Staphylococcus aureus craniotomy infection
Source: JCI Insight. 2025 Feb 24;10(4):e183327. doi: 10.1172/jci.insight.183327 (PMC11949042; doi:10.1172/jci.insight.183327)
Supplement: Supplemental data [file jciinsight-10-183327-s218.pdf]

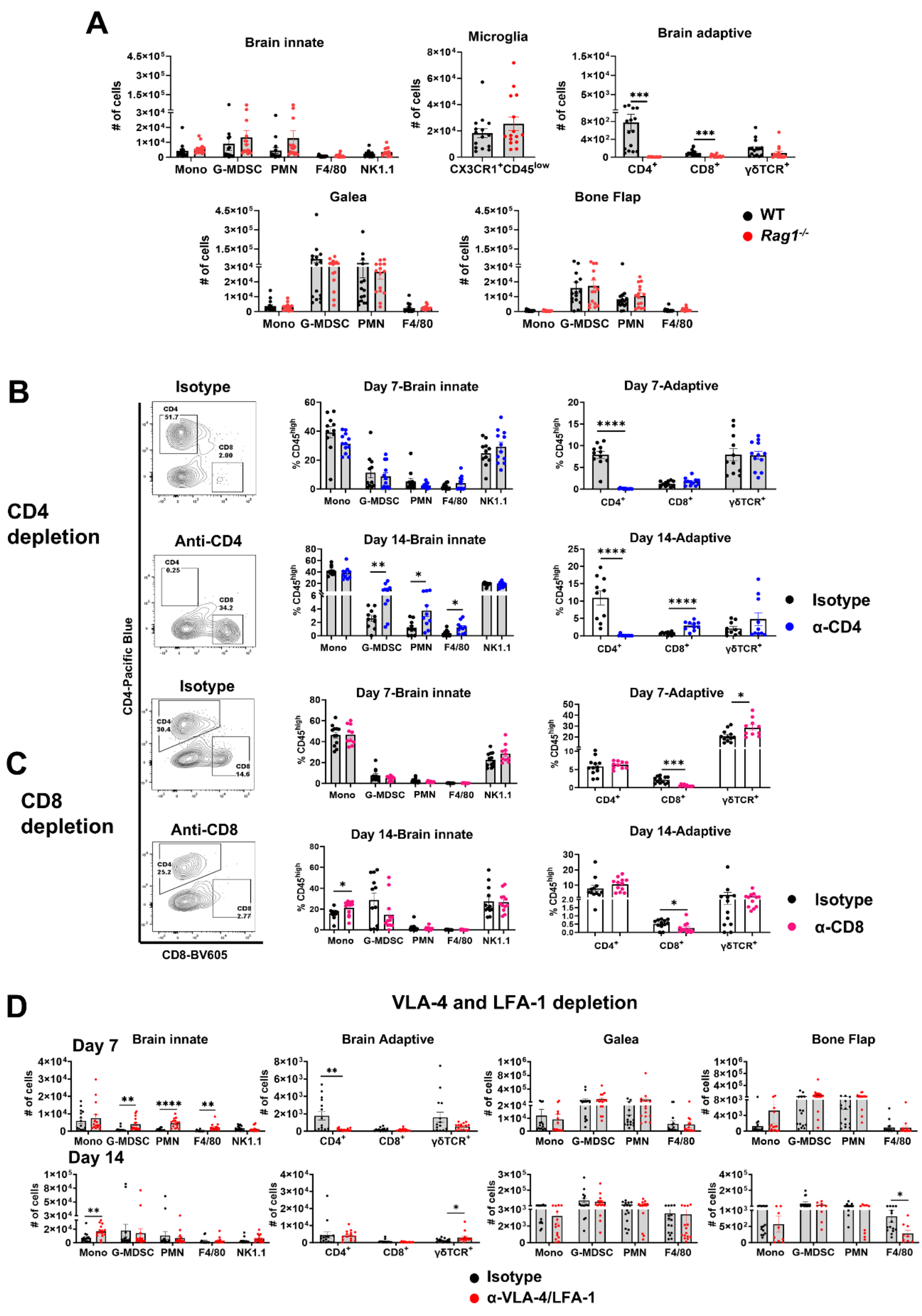
A
Brain innate
Microglia
Brain adaptive
# of cells
Mono
G-MDSC
PMN
F4/80
NK1.1
CX3CR1+CD45low
CD4+
CD8+
γδTCR+
Galea
Bone Flap
WT
Rag1-/-
B
CD4 depletion
Isotype
Anti-CD4
Day 7-Brain innate
Day 7-Adaptive
Day 14-Brain innate
Day 14-Adaptive
% CD45high
α-CD4
CD4-Pacific Blue
C
CD8 depletion
Anti-CD8
α-CD8
CD8-BV605
D
VLA-4 and LFA-1 depletion
Day 7
Day 14
Brain Adaptive
Isotype
α-VLA-4/LFA-1

Supplemental Fig. 1. Adaptive immunity influences leukocyte recruitment during *S. aureus* craniotomy infection. (**A**) Absolute counts of leukocyte infiltrates in the brain, galea and bone flap of WT vs *Rag1*$^{-/-}$ animals (n= 15/group) at day 14 post-infection. Bone flaps in some animals could not be recovered, resulting in smaller numbers in these groups. Data represent mean ± SEM combined from three independent experiments. Contour plots depicting (**B**) CD4^{+} and (**C**) CD8^{+} T cell depletion efficiency along with quantification of leukocyte infiltrates in the brain at days 7 and 14 post-infection (n=10-12/group). Data combined from two independent experiments. (**D**) Absolute counts of leukocyte infiltrates in the brain, galea and bone flap of mice receiving VLA-4 and LFA-1 or isotype control antibody at day 14 post-infection (n= 15/group). Data represents mean ± SEM combined from three independent experiments. *$p < 0.05$; **$p < 0.01$; ***$p < 0.001$; ****$p < 0.0001$, unpaired two-tailed Student's *t*-test.

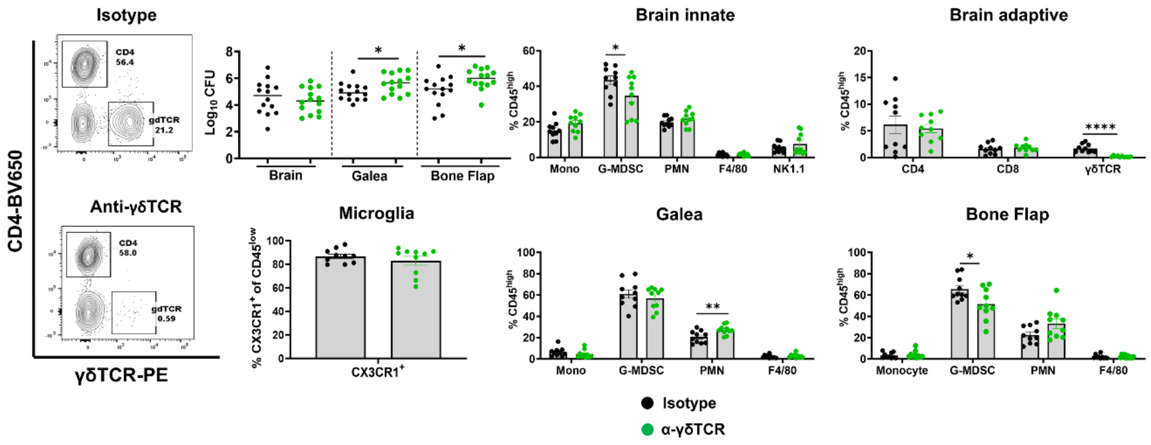

Supplemental Fig. 2. γδ T cells play a minor role during *S. aureus* craniotomy infection. Bacterial burdens and immune cell populations were quantified at day 14 post-infection in WT mice receiving anti-ɣδ TCR or isotype-matched control antibody (n=10-14 mice/group). Data combined from 2-3 independent experiments. *, $p < 0.05$; **, $p < 0.01$; ****$p < 0.0001$, unpaired two-tailed Student's *t*-test. $CD4^+$ and ɣδ T cells were gated from live $CD3^+CD45^+$ cells to validate ɣδ T cell depletion.

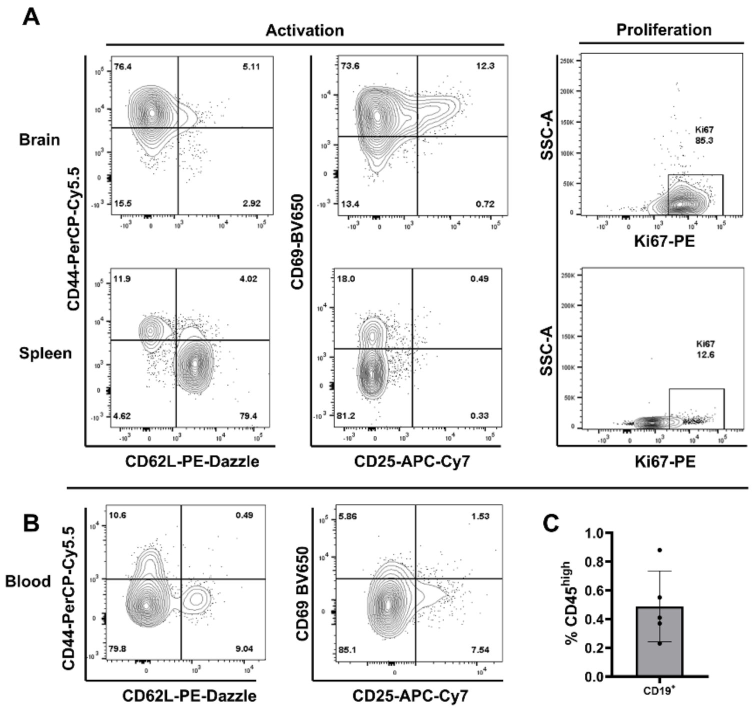

Supplemental Fig. 3. CD4⁺ T cells infiltrating the brain during *S. aureus* craniotomy infection are activated and proliferative. CD44, CD62L, CD25, CD69, and Ki67 expression in CD4⁺ T cells from the (**A**) brain and spleen or (**B**) blood of *S. aureus* infected WT mice. Brain and blood samples were pooled from 5 mice to recover sufficient numbers of CD4⁺ T cells for analysis, whereas 1 spleen sample was used. (**C**) Quantification of CD19⁺ B cell infiltrates in the brains of WT mice (n=5 individual animals) reported as the percentage of total $CD45^{hi}$ infiltrates.

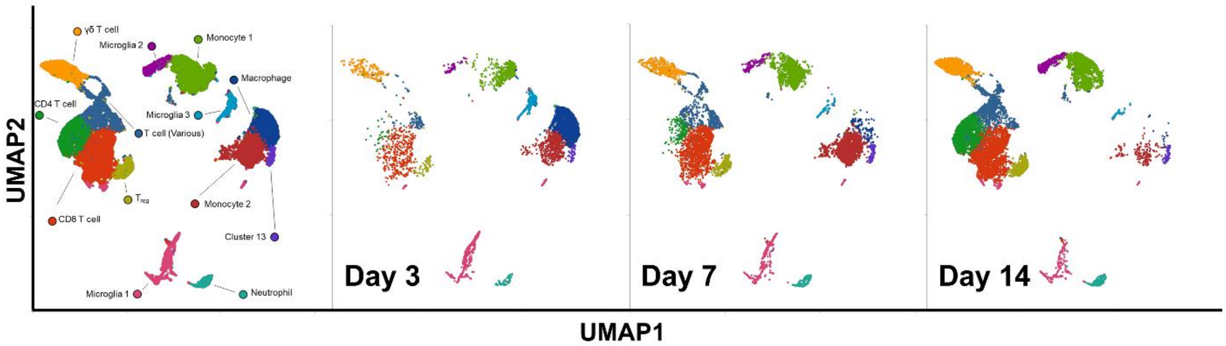

Supplemental Fig. 4. scRNA-seq analysis of cells recovered from the brain following CD3 enrichment. Aggregated UMAPs depicting various $CD3^+$ T cell and myeloid clusters in the brains of WT mice across each time point related to **Figure 3**.

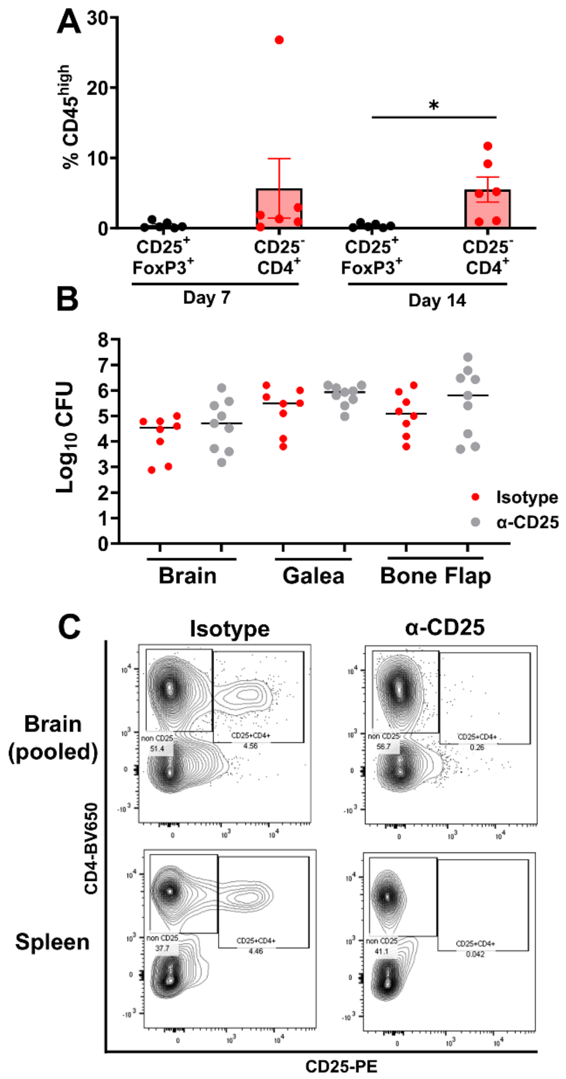

Supplemental Fig. 5. Tregs do not impact craniotomy infection. (**A**) The percentage of CD4$^+$CD25$^-$ T cells and CD4$^+$CD25$^+$FoxP3$^+$ Treg cells infiltrating the brain at days 7 and 14 post-infection (n=6 mice/group). *$p < 0.05$, unpaired two-tailed Student's *t*-test. (**B**) WT mice received anti-CD25 or isotype-matched control antibody, whereupon bacterial burdens were assessed at day 14 post-infection (n=8-9 mice/group combined from two independent experiments). (**C**) Contour plots depicting CD4$^+$CD25$^+$ T cell depletion efficiency in the brain and spleen at day 14 post-infection. T cells were pooled from 5 mice to obtain sufficient numbers for analysis.

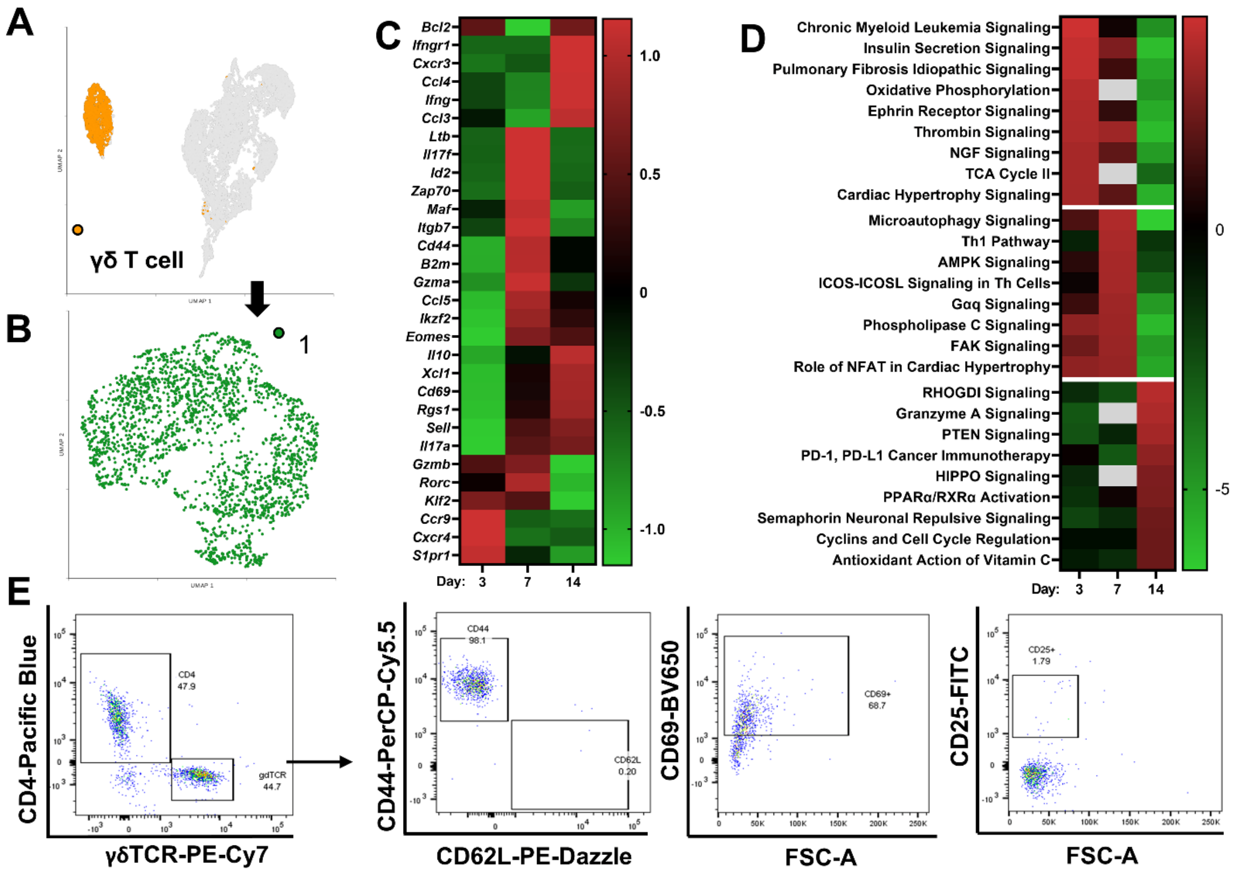

Supplemental Fig. 6. Brain γδ T cell infiltrates during *S. aureus* craniotomy infection display an activated phenotype. (**A, B**) scRNA-seq UMAP of the γδ T cell cluster in the brain aggregated across days 3, 7, and 14 post-infection with (**C**) genes and (**D**) top significantly expressed pathways for each γδ T cell cluster segregated according to time point. (**E**) $CD3^+$ cells were pooled from the brains of 5 infected mice at day 14 post-infection to assess CD44, CD62L, CD25, and CD69 expression on γδ TCR^+ cells.

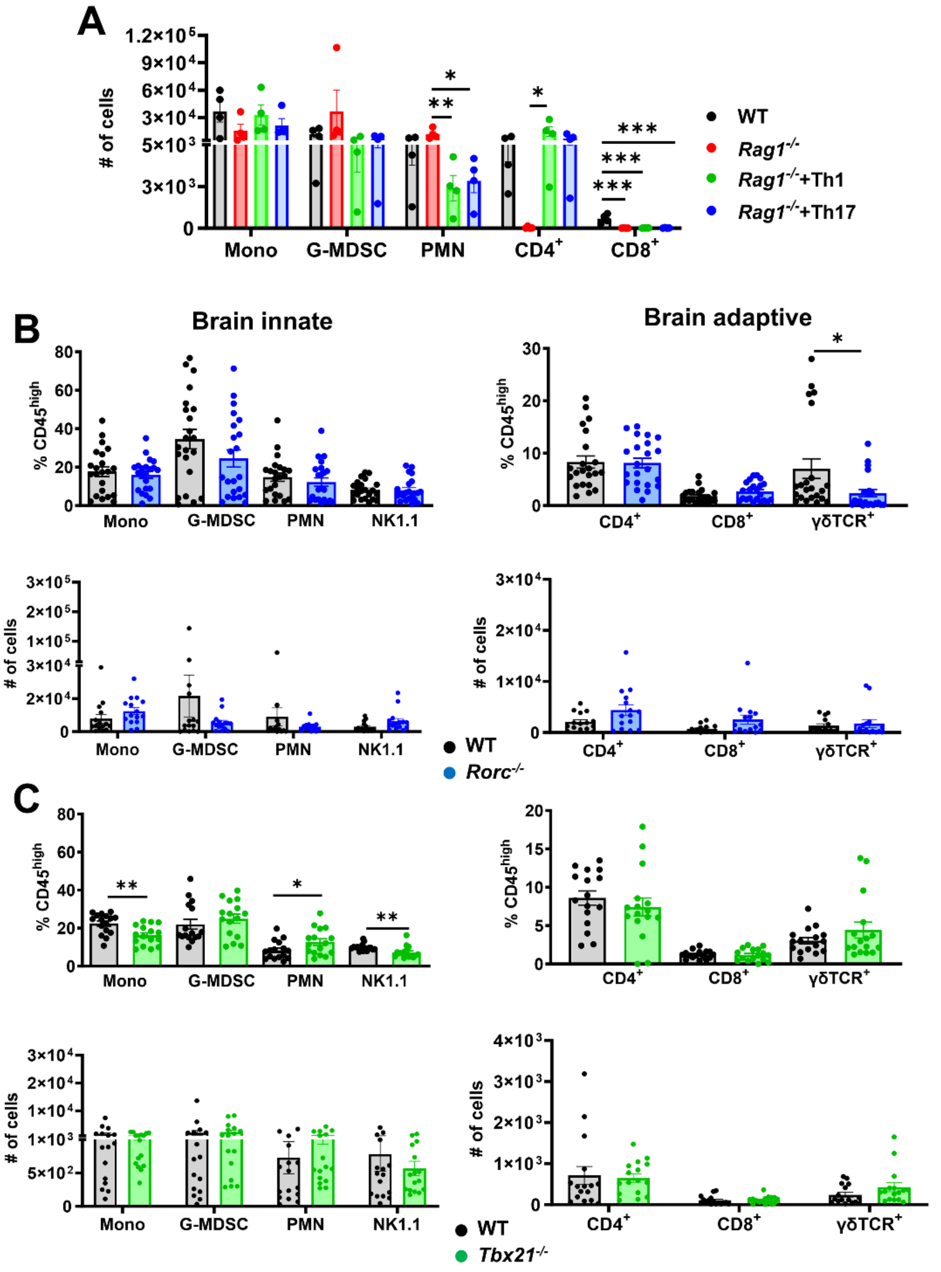

Supplemental Fig. 7. Th1 and Th17 responses do not significantly impact leukocyte recruitment to the brain during *S. aureus* craniotomy infection.

(**A**) Absolute counts of leukocyte infiltrates at day 14 post-infection in the brains of WT, *Rag1*$^{-/-}$, and *Rag1*$^{-/-}$ mice receiving either Th1 or Th17 cells (n=4/group). Results shown are representative of 3 independent experiments. Immune populations in the brains of (**B**) *Rorc*$^{-/-}$ and (**C**) *Tbx21*$^{-/-}$ mice at day 14 following craniotomy infection reported as percentage of CD45^{+}

cells and absolute counts. Results in (**B and C**) are combined from three independent experiments (n=15-22 mice/group). *$p < 0.05$; **$p < 0.01$; ***$p < 0.001$, unpaired two-tailed Student's *t*-test.

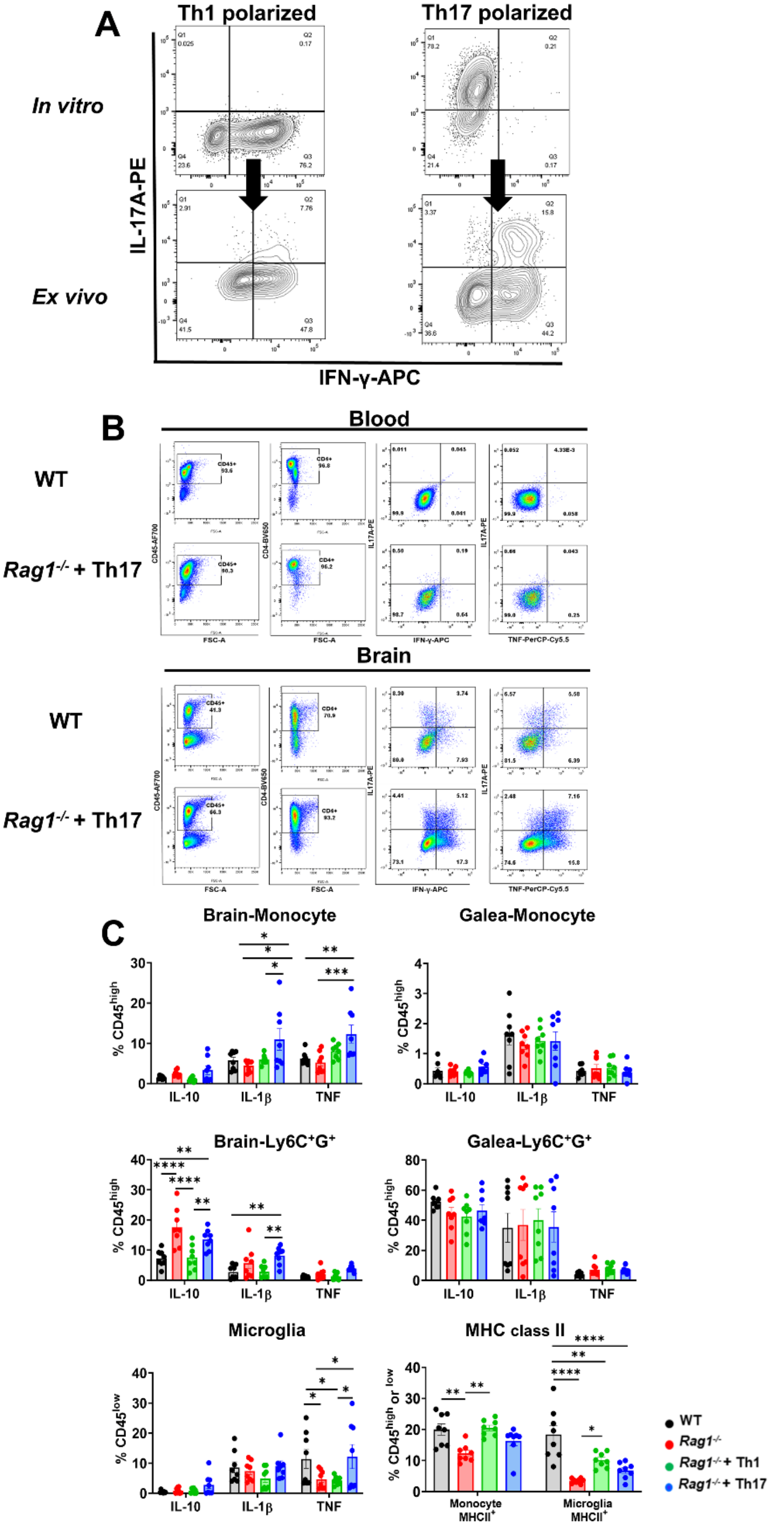

Supplemental Fig. 8. CD4⁺ T cell-mediated responses are typified by a dominant IFN-γ signature during craniotomy infection. (**A**) Cytokine profiles of CD4⁺ Th1 and Th17 cells prior

to adoptive transfer (from *in vitro* cultures) and after recovery from pooled brains (n=5) of *Rag1*$^{-/-}$ mice at day 14 post-infection. (**B**) *Ex vivo* cytokine staining of $CD4^{+}$ T cells recovered at day 14 post-infection from the blood and brains of WT and *Rag1*$^{-/-}$ mice following Th17 cell adoptive transfer. Samples were pooled from 5 mice to obtain sufficient numbers for analysis. (**C**) Cytokine production and MHC class II expression in innate immune cell populations at day 14 post-infection in the brain and galea of WT, *Rag1*$^{-/-}$, and *Rag1*$^{-/-}$ mice receiving Th1 or Th17 cells (n=8 mice/group). *, $p < 0.05$; **, $p < 0.01$; ***, $p < 0.001$; ****, $p < 0.0001$; Two-way ANOVA with Tukey's correction.

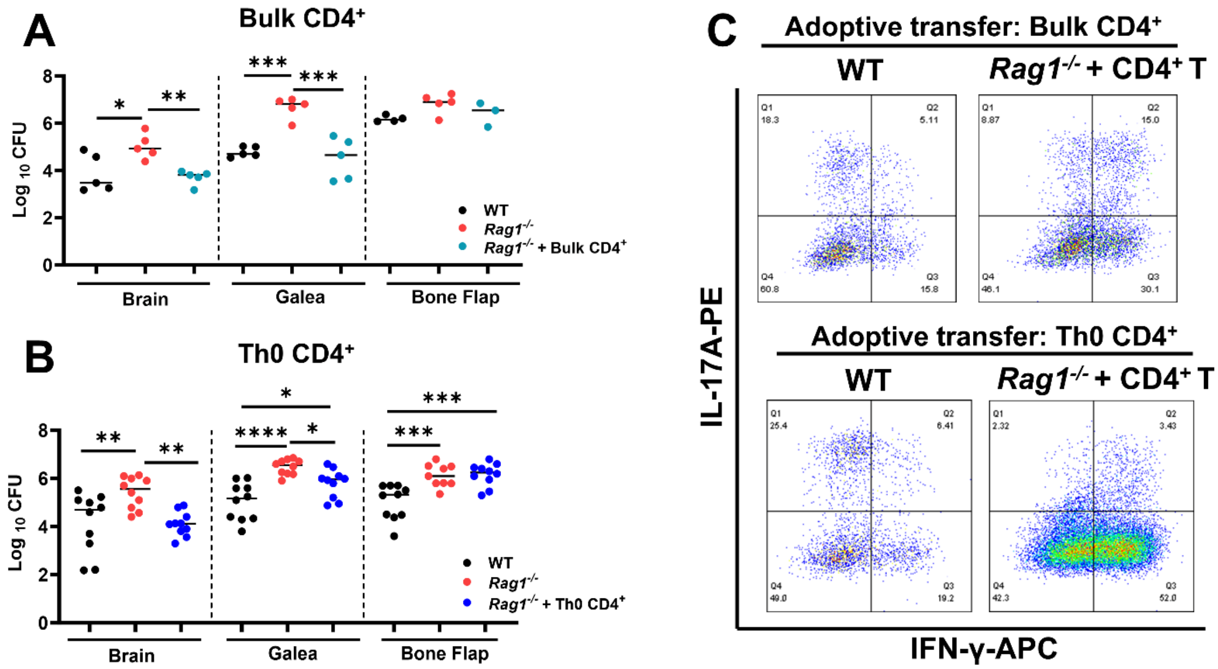

Supplemental Fig. 9. Non-polarized CD4⁺ T cells acquire an IFN-γ phenotype *in vivo* to mediate protection during craniotomy infection. (**A**) Bulk CD4⁺ (n= 5 mice/group) or (**B**) naïve CD4⁺ (Th0) T cells (n=10 mice/group combined from 2 independent experiments) were adoptively transferred into *Rag1*$^{-/-}$ mice at day -1 prior to *S. aureus* craniotomy infection, whereupon bacterial burden was quantified at day 14 post-infection in the brain, galea and bone flap. Bone flaps in some animals could not be recovered, resulting in smaller numbers in these groups. *, $p < 0.05$; **, $p < 0.01$; ***, $p < 0.001$; ****, $p < 0.0001$; One-way ANOVA with Tukey's correction. (**C**) IFN-γ and IL-17A production by CD4⁺ T cells recovered from brains of WT and *Rag1*$^{-/-}$ mice following adoptive transfer of bulk CD4⁺ or Th0 cells at day 14 post-infection. Leukocytes were pooled from the brains of 5 animals/group to obtain sufficient T cell numbers for analysis.

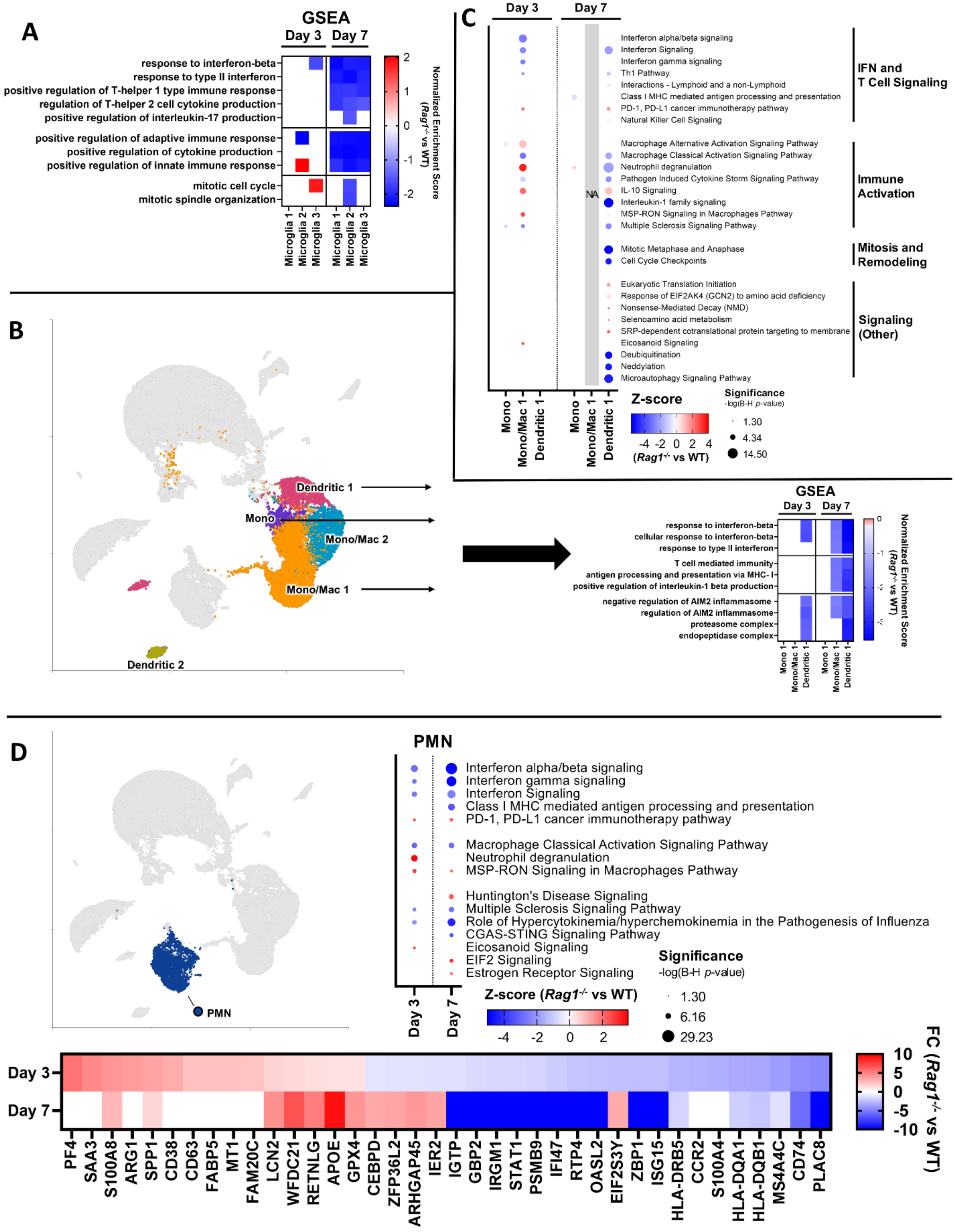
A
GSEA
Day 3
Day 7
response to interferon-beta
response to type II interferon
positive regulation of T-helper 1 type immune response
regulation of T-helper 2 cell cytokine production
positive regulation of interleukin-17 production
positive regulation of adaptive immune response
positive regulation of cytokine production
positive regulation of innate immune response
mitotic cell cycle
mitotic spindle organization
Microglia 1
Microglia 2
Microglia 3
Normalized Enrichment Score (Rag1-/- vs WT)
B
Dendritic 1
Mono
Mono/Mac 2
Mono/Mac 1
Dendritic 2
C
Interferon alpha/beta signaling
Interferon Signaling
Interferon gamma signaling
Th1 Pathway
Interactions - Lymphoid and a non-Lymphoid
Class I MHC mediated antigen processing and presentation
PD-1, PD-L1 cancer immunotherapy pathway
Natural Killer Cell Signaling
IFN and T Cell Signaling
Macrophage Alternative Activation Signaling Pathway
Macrophage Classical Activation Signaling Pathway
Neutrophil degranulation
Pathogen Induced Cytokine Storm Signaling Pathway
IL-10 Signaling
Interleukin-1 family signaling
MSP-RON Signaling in Macrophages Pathway
Multiple Sclerosis Signaling Pathway
Immune Activation
Mitotic Metaphase and Anaphase
Cell Cycle Checkpoints
Mitosis and Remodeling
Eukaryotic Translation Initiation
Response of EIF2AK4 (GCN2) to amino acid deficiency
Nonsense-Mediated Decay (NMD)
Selenoamino acid metabolism
SRP-dependent cotranslational protein targeting to membrane
Eicosanoid Signaling
Deubiquitination
Neddylation
Microautophagy Signaling Pathway
Signaling (Other)
NA
Mono
Mono/Mac 1
Dendritic 1
Z-score
(Rag1-/- vs WT)
Significance
-log(B-H p-value)
1.30
4.34
14.50
GSEA
response to interferon-beta
cellular response to interferon-beta
response to type II interferon
T cell mediated immunity
antigen processing and presentation via MHC-I
positive regulation of interleukin-1 beta production
negative regulation of AIM2 inflammasome
regulation of AIM2 inflammasome
proteasome complex
endopeptidase complex
D
PMN
Interferon alpha/beta signaling
Interferon gamma signaling
Interferon Signaling
Class I MHC mediated antigen processing and presentation
PD-1, PD-L1 cancer immunotherapy pathway
Macrophage Classical Activation Signaling Pathway
Neutrophil degranulation
MSP-RON Signaling in Macrophages Pathway
Huntington's Disease Signaling
Multiple Sclerosis Signaling Pathway
Role of Hypercytokinemia/hyperchemokinemia in the Pathogenesis of Influenza
CGAS-STING Signaling Pathway
Eicosanoid Signaling
EIF2 Signaling
Estrogen Receptor Signaling
Z-score (Rag1-/- vs WT)
Significance
-log(B-H p-value)
1.30
6.16
29.23
PF4 SAA3 S100A8 ARG1 SPP1 CD38 CD63 FABP5 MT1 FAM20C LCN2 WFDC21 RETNLG APOE GPX4 CEBPD ZFP36L2 ARHGAP45 IER2 IGTP GBP2 IRGM1 STAT1 PSMB9 IFI47 RTP4 OASL2 EIF2S3Y ZBP1 ISG15 HLA-DRB5 CCR2 S100A4 HLA-DQA1 HLA-DQB1 MS4A4C CD74 PLAC8
FC (Rag1-/- vs WT)

Supplemental Fig. 10. Adaptive immunity promotes innate immune cell activation during craniotomy infection. scRNA-seq was performed on $CD45^+$ cells recovered from the brains of WT and *Rag1*$^{-/-}$ mice at days 3 and 7 following *S. aureus* craniotomy infection. (**A**) IPA analysis depicting the top significant pathways in various microglial clusters and (**B**) UMAPs highlighting various monocyte/macrophage and dendritic cell clusters along with top significant pathways. (**C**) Pathway analyses for each cluster in the UMAP shown in panel (**B**). (**D**) Top significant pathways for the single PMN cluster in the brain are presented along with molecules predicted to be upregulated (red), downregulated (blue), or undetermined (grey).

Feature ID	FDR step up (*Rag1*$^{-/-}$ vs WT)	Fold change (*Rag1*$^{-/-}$ vs WT)	LSMean(*Rag1*$^{-/-}$) (*Rag1*$^{-/-}$ vs WT)	LSMean(WT) (*Rag1*$^{-/-}$ vs WT)
Plac8	0.00	-4.61	16.96	78.18
Cd74	0.00	-4.41	41.52	183.29
Ms4a4c	0.00	-3.86	13.22	50.99
H2-Ab1	0.00	-3.46	8.15	28.22
H2-Aa	0.00	-3.39	4.82	16.35
S100a4	0.00	-3.17	113.42	359.01
Ccr2	0.00	-3.03	105.76	320.74
H2-Eb1	0.00	-2.91	4.33	12.62
Isg15	0.00	-2.49	6.31	15.71
Zbp1	0.00	-2.48	10.04	24.88
Eif2s3y	0.00	-2.44	1.43	3.49
Oasl2	0.00	-2.43	6.21	15.09
Ly6c2	0.00	-2.41	7.30	17.63
Ifi203	0.00	-2.39	5.60	13.40
Ighm	0.00	-2.35	2.40	5.65
Ramp1	0.00	-2.33	5.97	13.90
Irf7	0.00	-2.31	4.29	9.92
Rtp4	0.00	-2.31	7.83	18.08
Cx3cr1	0.00	-2.21	52.72	116.59
Plbd1	0.00	-2.20	53.55	117.89
Slfn5	0.00	-2.16	28.39	61.46
Slfn1	0.00	-2.13	7.01	14.91
H2-DMb1	0.00	-2.10	9.51	20.01
Epsti1	0.00	-2.08	28.13	58.46
Tmem176b	0.00	-2.05	9.11	18.63
Ddx3y	0.00	-2.04	1.28	2.61
Ifi211	0.00	-2.01	4.58	9.19
Ifi47	0.00	-2.00	2.78	5.57
Chil3	0.00	2.01	61.70	30.76
Syngr1	0.00	2.05	38.87	19.00
Cxcl2	0.00	2.05	4.43	2.16
Cd93	0.00	2.05	60.37	29.42
Slc48a1	0.00	2.06	31.07	15.05
Nrp2	0.00	2.07	23.10	11.18
Lrg1	0.00	2.08	17.70	8.50
Sdc4	0.00	2.14	19.87	9.29
S100a9	0.00	2.25	9.59	4.27
F13a1	0.00	2.28	186.81	82.05
Hmox1	0.00	2.29	370.18	161.91
Bnip3	0.00	2.31	22.54	9.74
Fam20c	0.00	2.32	14.76	6.36
Mt1	0.00	2.40	385.39	160.52
Fabp5	0.00	2.45	170.77	69.69
Cd63	0.00	2.47	133.36	54.09
Cd38	0.00	3.23	48.09	14.87
Spp1	0.00	3.58	270.06	75.42
Arg1	0.00	3.82	144.13	37.73
S100a8	0.00	4.14	31.58	7.63
Saa3	0.00	4.63	24.07	5.20
Pf4	0.00	5.46	83.45	15.27

Supplemental Fig. 11. Comparison of transcriptomic profiles of monocyte/macrophage cluster 1 in *Rag1*$^{-/-}$ vs WT mice during craniotomy infection. scRNA-seq was performed on CD45^{+} cells recovered from the brains of WT and *Rag1*$^{-/-}$ mice at days 3 and 7 following *S.*

aureus craniotomy infection. Results depict the 50 genes identified as significantly differentially expressed in monocyte/macrophage cluster 1 (Mono/Mac 1) between *Rag1*$^{-/-}$ vs WT mice at day 3 post-infection, related to **Figure 8**.

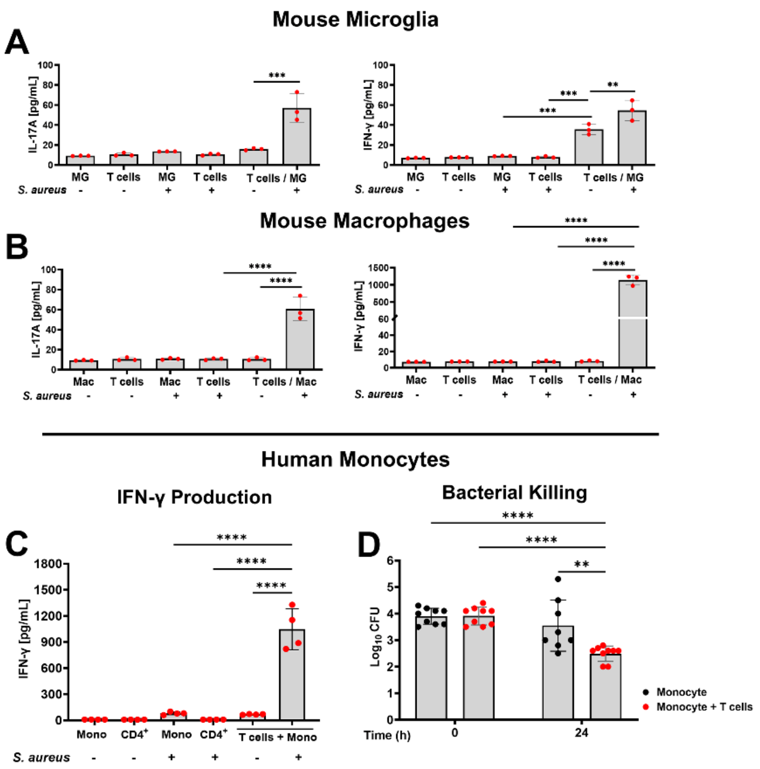

Supplemental Fig. 12. CD4⁺ T cell-innate immune cell crosstalk augments responses to *S. aureus*. (**A**) Primary mouse microglia and (**B**) macrophages were exposed to live *S. aureus* at a multiplicity of infection (MOI) of 10:1 (bacteria:immune cell) for 1 h followed by gentamicin treatment to kill remaining extracellular bacteria and co-cultured with mouse $CD4^+$ cells at a 1:1 ratio for 24 h, whereupon IFN-γ and IL-17A levels were quantified by cytometric bead array. Data is from one representative experiment (n= 3 biological replicates). (**C-D**) Human peripheral blood monocytes ($CD14^+CD16^+$) were treated with live *S. aureus* for 1 h at a MOI of 1:1 and co-cultured with autologous human $CD4^+$ T cells to evaluate (**C**) IFN-γ production by cytometric bead array and (**D**) bacterial killing using a gentamicin protection assay. **, $p < 0.01$; ***, $p < 0.001$; ****, $p < 0.0001$; (**A-C**) One-way or (**D**) Two-way ANOVA with Tukey's correction.

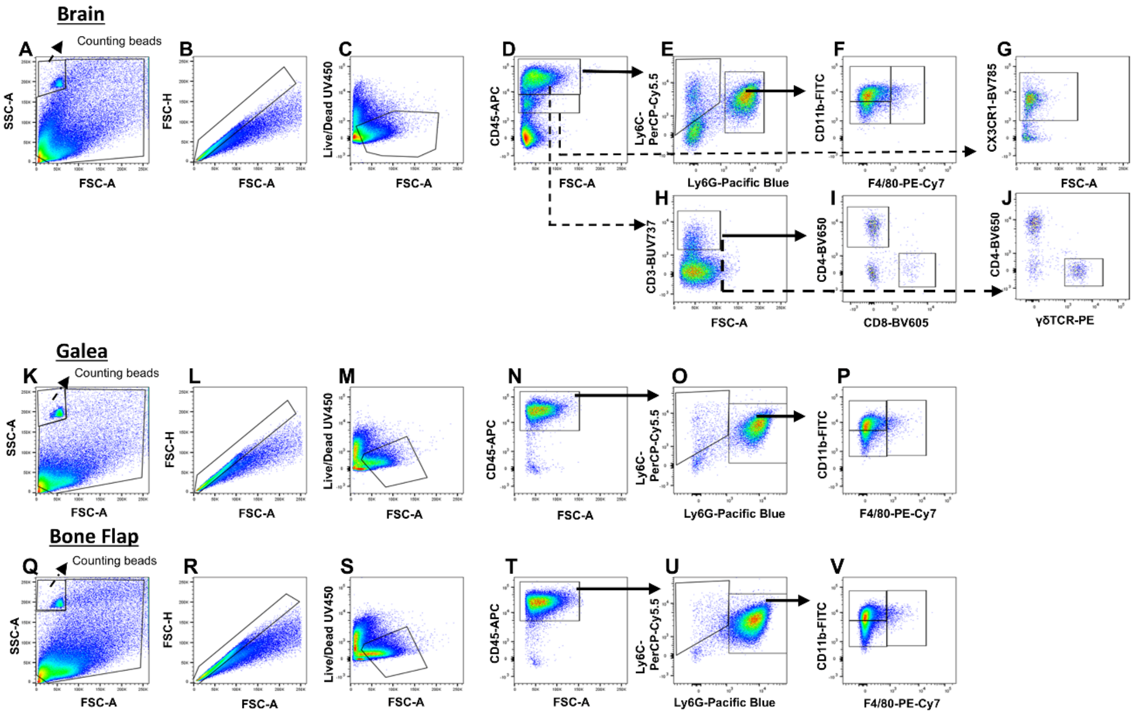

Supplemental Fig. 13. Flow cytometry gating strategy. From the (**A**, **K, Q**) total events, (**B**, **L, R**) single cells were gated using FSC-A vs. FSC-H, followed by (**C**, **M, S**) exclusion of dead cells. For the innate immune panel, (**D, N or T**) live, CD45^{high} leukocytes were separated into (**E, O, U**) Ly6G^{-}Ly6C^{+} monocytes vs. Ly6G^{+}Ly6C^{+} cells, which were further identified as (**F, P, V**) G-MDSCs (CD11b^{high}Ly6G^{+}Ly6C^{+}F4/80^{-}) and neutrophils (CD11b^{low}Ly6G^{+}Ly6C^{+}F4/80^{-}) based on CD11b and F4/80 expression. Microglia were defined as (**D, G**) CD45^{low} vs. FSC and CX3CR1^{+}. Adaptive immune cells were subjected to the same initial steps (**H-J**) as described for the innate immune panel, where (**D**) live, CD45^{high} leukocytes were separated into (**H**) CD3^{+} cells that were gated on (**I**) CD4^{+} and CD8^{+} T cells and (**J**) γδ T cells (γδ TCR^{+}).

Supplemental Table 1. Antibodies used in this study

ANTIBODY	SOURCE	IDENTIFIER
**Flow cytometry**		
Anti-mouse CD45-APC	BioLegend	RRID:AB_312977
Anti-mouse CD45-Alexa Fluor 700	BioLegend	RRID:AB_493715
Anti-mouse CD45-BUV805	BioLegend	RRID:AB_2872789
Anti-mouse Ly6G-PE	BioLegend	RRID:AB_1186099
Anti-mouse Ly6G-Pacific Blue	BioLegend	RRID:AB_2251161
Anti-mouse CX3CR1-BV785	BioLegend	RRID:AB_2565938
Anti-mouse CD11b-FITC	BioLegend	RRID:AB_312789
Anti-mouse CD11b-Alexa Fluor 700	BioLegend	RRID:AB_493705
Anti-mouse F4/80-BV510	BioLegend	RRID:AB_2562622
Anti-mouse F4/80-PE-Cy7	BioLegend	RRID:AB_893478
Anti-mouse Ly6C-APC-Cy7	BioLegend	RRID:AB_1727555
Anti-mouse Ly6C-PerCP-Cy5.5	BD	RRID:AB_1727558
Anti-mouse CD3-BUV737	BD	RRID:AB_2870130
Anti-mouse CD3-Pacific Blue	BioLegend	RRID:AB_493645
Anti-mouse CD3-APC	BioLegend	RRID:AB_2561456
Anti-mouse CD3-FITC	BioLegend	RRID:AB_312661
Anti-mouse CD4-PacificBlue	BioLegend	RRID:AB_493374
Anti-mouse CD4-BV650	BioLegend	RRID:AB_2562529
Anti-mouse CD8a-BV605	BioLegend	RRID:AB_2562609
Anti-mouse CD8a-Alexa Fluor 700	BioLegend	RRID:AB_493702
Anti-mouse CD8a-PE-Cy7	BioLegend	RRID:AB_312760
Anti-mouse γδTCR-PE	BioLegend	RRID:AB_313832
Anti-mouse γδTCR-BV510	BioLegend	RRID:AB_2563534
Anti-mouse γδTCR-PE-Cy7	BioLegend	RRID:AB_11204423
Anti-mouse NK1.1-APC-Cy7	BioLegend	RRID:AB_830871
Anti-mouse FoxP3-AF647	BioLegend	RRID:AB_439750
Anti-mouse CD19-Alexa Fluor 700	BioLegend	RRID:AB_493735
Anti-mouse MHC-II (I-A/I-E)-BV605	BioLegend	RRID:AB_2565894
Anti-mouse CD44-PerCP-Cy5.5	BioLegend	RRID:AB_2076204
Anti-mouse CD62L-PE-Dazzle	BioLegend	RRID:AB_2566162
Anti-mouse CD25-FITC	BioLegend	RRID:AB_312855
Anti-mouse CD25-APC-Cy7	BioLegend	RRID:AB_830744
Anti-mouse CD69-BV650	BioLegend	RRID:AB_2616934
Anti-mouse Ki67-PE	BioLegend	RRID:AB_2561525
Anti-mouse IFN-γ-APC	BioLegend	RRID:AB_315403
Anti-mouse IL-17A-PE	BioLegend	RRID:AB_315463
Anti-mouse IL-10-PE-Cy7	BioLegend	RRID:AB_11150582
Anti-mouse TNF-PerCP-Cy5.5	BioLegend	RRID:AB_961434
Anti-mouse IL-1β-APC eFluor780	ThermoFisher	RRID:AB_2573996
**In vivo depletion**		
InVivo MAb anti-mouse CD4	BioXCell	RRID:AB_1107636

InVivo MAb anti-mouse CD8α	BioXCell	RRID:AB_1107671
InVivoMAb rat IgG2b isotype control, anti-keyhole limpet hemocyanin	BioXCell	RRID:AB_1107780
InVivoMAb rat IgG2a isotype control, anti-trinitrophenol	BioXCell	RRID:AB_1107769
InVivo MAb anti-mouse CD25	BioXCell	RRID:AB_1107619
InVivoMAb rat IgG1 isotype control, anti-horseradish peroxidase	BioXCell	RRID:AB_1107775
InVivo MAb anti-mouse TCR γ/δ	BioXCell	RRID:AB_1107751
InVivoMAb polyclonal Armenian hamster IgG	BioXCell	RRID:AB_1107773
InVivo MAb anti-mouse/human VLA-4	BioXCell	RRID:AB_1107657
InVivo MAb anti-mouse LFA-1α	BioXCell	RRID:AB_1107574
InVivo Mab anti-mouse IL-17A	BioXCell	RRID:AB_10950102
InVivoPlus mouse IgG1 isotype control, unknown specificity	BioXCell	RRID:AB_1107784
InVivo MAb anti-mouse CD119	BioXCell	RRID:AB_1107576

Supplemental Table 2. Demographics of human subjects with craniotomy infection

Demographics					Pathogen		Specimens Collected			
**Procedure**	**Pat. #**	**Sex**	**Age**	**Race**	**Pathogen**	**Gram**	**Blood**	**Galea**	**Flap**	**ICT**
Craniotomy	7	F	51	Caucasian	*S. epidermidis*	(+)	✓	✓	✓	✓
Craniotomy	11	M	39	Caucasian	*C. acnes*	(+)	✓	✓	✓	
Craniotomy	14	F	44	Caucasian	*C. acnes*	(+)	✓	✓	✓	
Craniotomy	15	M	45	Caucasian	*S. aureus*	(+)	✓	✓	✓	
